# Health literacy of adults from primary care with type 2 diabetes *mellitus*: a cross-sectional study

**DOI:** 10.1590/1980-220X-REEUSP-2024-0338en

**Published:** 2025-05-16

**Authors:** Robson Giovani Paes, Isabella Bueno Fusculim, Luciane Lachouski, Ingrid Marcela Pinto Gariba de Andrade, Shirley Boller, Maria de Fátima Mantovani

**Affiliations:** 1 Universidade Federal do Paraná, Curitiba, PR, Brazil.

**Keywords:** Nursing, Diabetes Mellitus, Health Literacy, Primary Health Care

## Abstract

**Objective:**

To relate health literacy of adults from primary health care with type 2 diabetes mellitus and sociodemographic and clinical variables.

**Method:**

Descriptive analytical cross-sectional study with 169 participants aged 18 to 65 with type 2 diabetes mellitus, from six primary care units in the Metropolitan Region of Curitiba, Paraná, Brazil. Data collection took place from July 2023 to March 2024, and a sociodemographic and clinical questionnaire was used, as well as subscales six and nine of the *Health Literacy Questionnaire,* Brazilian version. Data were analyzed descriptively and using Student’s t-test, Mann-Whitney, Anova, Kruskal-Wallis, and Spearman tests.

**Results:**

Of the 169 participants, the mean age was 56.5 ± 6.2 years. There was a significant association between the ability to interact with health professionals and male gender (*p* = 0.057), fasting blood glucose ≤ 130 mg/dL (*p* = 0.033), and between education and understanding health information and knowing what to do (*p* = <0.001). A weak correlation was found between understanding health information and knowing what to do and education and family income (*r* = 0.50 and 0.35, respectively) and significant value with glycated hemoglobin (*p* = 0.031).

**Conclusion:**

There was a relationship between health literacy and male gender, education, family income, and glycemic values. These results can contribute to the development of educational actions aimed at strengthening health literacy.

## INTRODUCTIOn

Health literacy (HL) is a potential social determinant of health^(1)^ that expresses the degree of capacity the individual has to receive, process, and understand the basic information and services required to make appropriate decisions regarding their health status^(1,2)^. It is defined as the knowledge, motivation, and skills to access, evaluate, and apply information in the field of disease prevention, health care, and health promotion to improve or maintain quality of life and decision-making autonomy^(2)^.

HL can also be conceptualized as a set of individual and social skills, through information sharing among professionals, users, and systems, in strengthening timely judgment for decision-making in health^(3)^. HL levels analysis allows identifying the degree of involvement of the individual with the health-disease process, their recognition of risk factors, access to information and services, and the possibility of creating actions for active participation in health-related care^(4)^.

HL analysis is in line with the preventive and surveillance measures for chronic non-communicable diseases and conditions, presented in the strategic action plan of the Brazilian Ministry of Health (MS), which advocates the importance of people’s knowledge about risk factors, the access to public goods and services, and the guarantee of information rights, which allow for choices that are favorable to health^(5)^.

When considering chronic non-communicable diseases, diabetes *mellitus* (DM) stands out as an important public health problem, due to its prevalence and complications^(6)^. Type 2 diabetes mellitus (T2DM) accounts for about 90-95% of cases of the disease and has as triggering factors family history, difficulty in controlling weight, and sedentary lifestyle habits, leading to high rates of micro and macrovascular complications, such as stroke, acute myocardial infarction, amputations, retinopathy, neuropathy, and other problems^(6)^.

In 2021, Brazil was in sixth place in the worldwide incidence of the disease, with approximately 15 million people with DM in the age group of 20 to 79 years, with a projected increase of 7.5 million new cases by the year 2045^(7)^. This projection becomes worrying in the presence of complications typically common to T2DM, which may be related to low levels of HL and lack of knowledge about the disease, which may directly interfere with obtaining and understanding health information^(4)^.

These aspects were highlighted in a previous study carried out with 33 adults diagnosed with T2DM, registered in a health unit in the southern region of Brazil, to whom the instruments *Spoken Knowledge in Low Literacy Patients with Diabetes* (SKILLD) and *Eight-Item Health Literacy Assessment Tool* (HLAT-8) were applied to analyze knowledge about the disease and HL. After the educational nursing intervention, there was an increase in knowledge about DM (*p* = 0.001) correlated to HL (*p* = 0.001)^(4)^, and it can be inferred that the professional’s interaction and person-centered actions helped participants in the search for and understanding of health information and services.

In this regard, we sought to understand the ability of adults with T2DM to interact with health professionals and to understand health information and know what to do, based on a British study with 2,309 participants, which related subscales six and nine of the *Health Literacy Questionnaire* (HLQ) regarding the skills of interacting with professionals and understanding health information, with sociodemographic and clinical variables. The results showed that 19.4% of participants had difficulty reading and understanding written health information and 23.2% had difficulty discussing their concerns with health professionals due to their low social and educational status. It should be noted that the participants had at least one chronic disease^(8)^.

Therefore, it becomes essential to identify communication barriers, either due to the technically complex language used by the health professional, or due to incorrect or incomplete information received by the interlocutor, factors that can interfere with the fulfillment of health interventions, worsening of the clinical condition^(9)^, and the development of HL skills. It is revealed that people with T2DM with high levels of HL tend to have better adherence to drug treatment and lifestyle changes, with an outcome in glycemic control and mitigation of complications associated with the disease^(10)^.

In this regard, aiming at contributing to care focused on comprehensive assistance to people with T2DM in the context of Primary Health Care (PHC), based on the degree of HL, this study had as objective to relate health literacy with sociodemographic and clinical variables of adults from primary health care with type 2 diabetes mellitus.

## METHOD

### Design of Study

Quantitative, descriptive, analytical, sectional study that followed the recommendations of *Strengthening the Reporting of Observational studies in Epidemiology* (Strobe)^(11)^.

### Population, Local, and Selection Criteria

The study was conducted in six PHC units located in the Metropolitan Region of Curitiba, Paraná, Brazil. The municipality was among the ten most populous cities in the State, with approximately 232,212 inhabitants and a Municipal Human Development Index (IDHM) of 0.733, considered similar to that of the country. Its territorial distribution was 197.580 km^2^ with a population density of 1,175.28 inhabitants/km^2(12)^.

In the health sphere, the municipality had 25 PHC units, organized into three health districts, with approximately 11,278 users diagnosed with DM and registered in the Ministry of Health’s Hypertension and DM Program (Hiperdia).

The eligibility criteria were users of health services aged between 18 and 65 years, registered in the *Hiperdia* Program and with a confirmed diagnosis of T2DM. Users with cognitive and/or communication comorbidities, identified by the description in the electronic medical record, were excluded.

### Sample Definition and Recruitment

The study is part of a larger project that used population- based probability sampling by clusters. This sampling was planned based on the estimated number of inhabitants of the municipality, considering a national DM prevalence rate of 10.5% (95% confidence interval [CI]: 9.4% to 11.6%), an acceptable error of 5% and a heterogeneous sample (50/50). The number of participants per cluster was defined proportionally to the total number of registered users with DM in each unit, and in this subproject, the data refer to 25% (six PHC units), two from each health district.

Participants were selected using an online drawing application, using the names of all users on the Hiperdia Program lists, provided by the coordinators of the PHC units. This procedure ensured sample randomness, allowing subsequent access to medical records to identify and define eligible users. The lists were organized with the inclusion of substitutes, aiming at replacing volunteers in case of refusals or non-existent contacts.

The recruitment of selected users was carried out simultaneously with data collection, through telephone calls and/or standard text messages sent via the WhatsApp®️ application. Up to three contact attempts were made, on different days and times, to invite users to participate in the study. In cases of acceptance, a nursing consultation was scheduled, to be carried out at the health unit or at home, depending on the participant’s availability and preference. In total, 596 users were contacted: 381 did not answer calls or respond to messages, 46 refused to participate and 169 made up the final sample.

### Data Collection Instruments

Two instruments were used to collect data: a questionnaire containing sociodemographic and clinical variables and the Brazilian version of the HLQ (HLQ-Br). The questionnaire contained sociodemographic variables (age, sex, education, marital status and family income) and clinical variables (time since diagnosis of T2DM, glycated hemoglobin (HbA1c) tests and fasting blood glucose).

The HLQ-Br is a multidimensional instrument developed in Australia, which can be self-administered or applied in the form of an interview. It contains 44 questions distributed in nine subscales: 1 – Understanding and support from health professionals; 2 – Sufficient information to take care of health; 3 – Active health care; 4 – Social support for health; 5 – Evaluation of health information; 6 – Ability to interact actively with health professionals; 7 – Navigate the health system; 8 – Ability to find good health information; and 9 – Understand health information and know what to do. It has been validated for Brazilian Portuguese^(13,14)^.

The HLQ-Br is a tool that helps identify HL conditions. For this study, subscales 6 and 9 were selected, which address issues related to communication with health professionals and to understanding and following health instructions. Responses to these subscales follow a five-point Likert format, ranging from “always difficult (1)” to “always easy (5)”. The HLQ score was calculated based on the average of the items in each subscale.

### Data Collection

Data collection was carried out during the nursing consultation, lasting an average of 50 minutes, conducted by the main researcher and 10 research assistants, who received five hours of prior training, through a theoretical-practical course covering the topics of HL and DM.

A sociodemographic and clinical questionnaire was applied with the variables of age, sex, education, marital status, family income and time since diagnosis of T2DM, which were obtained through self-declaration. It should be noted that one participant did not know or did not want to report their monthly family income.

The HbA1c and fasting blood glucose test values were obtained from the participant’s medical records and were valid up to one year before the study. In cases where the biochemical variables were outside the established deadline, the tests were requested to be performed in laboratories accredited by the municipal health department, through the MS *Previne Brasil* Program. It is worth noting that the request for the collection of tests was delivered to 25 participants, but four did not perform them, reducing the sample size for this variable.

Subsequently, the two HLQ-Br subscales were applied in approximately 20 minutes. For participants with low vision and reading difficulties, the instrument was applied in the form of an interview.

### Data Analysis and Treatment

Data was entered into *Microsoft Excel*® spreadsheets by two researchers independently, with both spreadsheets being compared by the statistics professional to check for any inconsistencies, and the data were analyzed with the help of the IBM® computer program *Statistical Package for Social Sciences* (SPSS) v.29.0.0. The analysis of quantitative descriptive vari­ables (age, education, family income, time since diagnosis, HbA1c values and fasting blood glucose) was presented as mean and standard deviation (SD).

Categorical variables were described by absolute frequency and percentage, being age (45–59; 60–65 years), sex (male/female), marital status (single/married/separated or divorced/widowed), education (illiterate; ≤ 5; 6–9; 10–12 and > 12 years), family income (< 1,320; 1,320–3,960; > 3,960 reais per month), time from diagnosis (< 1; 1–5; 6–10 and > 10 years), HbA1c (< 7 and ≥ 7%), and fasting blood glucose (≤ 130 and > 130 mg/dL).

To analyze the association of the subscales of the HLQ-Br questionnaire with sociodemographic and categorical clinical variables with two classifications, the Student’s t-test for independent samples or the non-parametric Mann-Whitney test were used. Variables with more than two classifications were analyzed using the one-factor analysis of variance (ANOVA) model or the non-parametric Kruskal-Wallis test. For the correlation analysis between the subscales and the quantitative variables, Spearman’s Correlation Coefficients were estimated (*r*). The correlation values were interpreted as excellent (*r =* > 0.90), good (*r =* 0.75 to 0.90), moderate (*r* = 0.50 to 0.74), and weak (*r =* < 0.50). The p-values < 0.05 indicated statistical significance.

The results of subscales 6 and 9 of the HLQ-Br were presented in a specific and estimated measure, with a 95% CI. The reliability of the subscales for the adult population with T2DM was tested using Cronbach’s alpha coefficient (α), with a minimum value of 0.70 being considered.

### Ethical Aspects

The study was approved by the Human Research Ethics Committee of the Universidade Pública do Estado do Paraná under the consolidated opinion no. 6.138.731 and CAAE 68471723.6.0000.0102. For the development of the research, the requirements of the National Health Council (CNS) set out in Resolution No. 466/2012 were followed.

The HLQ was developed by Swinburne University of Technology, in Australia, which holds the intellectual property rights to the material. For its applicability in this study, the license was obtained under code L23009IS, and the authors’ recommendations for presenting the instrument’s questions were followed^(14)^.

## RESULTS

Of the 169 (100%) participants with T2DM, 58.0% (n = 98) were women, aged between 40 and 65 years (56.5 ± 6.2), average family income of R$ 4,032 ± 3,021 and 37.3% (n = 63), with education equal to or less than five years, and 75.1% (n = 127) were married and/or in a consensual union. Regarding clinicalvariables, 78.9% (n = 130) had HbA1c values ≥ 7%, 63.6% (n = 105) with fasting glucose > 130 mg/dL and 46.7% (n = 79) and had been diagnosed with T2DM more than 10 years ago (Table 1).

**Table 1:** Sociodemographic and clinical characterization of adults with Type 2 Diabetes Mellitus – Metropolitan Region of Curitiba, PR, Brazil, 2024.

Variables	n (%)	Mean ± SD
Age (years)		
40–59	106 (62.7)	56.5 ± 6.2
60–65	63 (37.3)	
(total n)	169 (100)	
Sex		
Female	98 (58.0)	
Male	71 (42.0)	
(total n)	169 (100)	
Marital status		
Single	15 (8.9)	
Married/consensual union	127 (75.1)	
Separated/divorced	19 (11.2)	
Widow(er)	8 (4.7)	
(total n)	169 (100)	
Education (years)		
Not literate	10 (5.9)	7.6 ± 4.7
≤ 5	63 (37.3)	
6–9	33 (19.5)	
10–12	41 (24.3)	
> 12	22 (13.0)	
(total n)	169 (100)	
Family income (reais)*		
< 1,320	17 (10.1)	4,032 ± 3,021
1.320–3.960	78 (46.4)	
> 3,960	73 (43.5)	
(total n)	168 (100)	
Diagnostic time (years)		
< 1	1 (0.6)	11.89 ± 8.6
1–5	48 (28.4)	
6–10	41 (24.3)	
> 10	79 (46.7)	
(total n)	169 (100)	
HbA1c (%)		
< 7	35 (71.4)	8.7 ± 2.1
≥ 7	130 (78.8)	
(total n)	165 (100)	
Fasting Blood Glucose (mg/dL)		
≤ 130	60 (36.4)	173.6 ± 81.7
> 130	105 (63.3)	
(total n)	165 (100)	

*Referring to the Brazilian minimum wage in July 2023.

Mean HL in subscale 6, that is, ability to actively interact with health professionals, was 3.67 ± 0.88 (95% CI: 3.53–3.80) and showed a significant association with the male sex (p = 0.057) and those who had fasting blood glucose values ≤ 130 mg/dL (p = 0.033) (Table 2).

**Table 2:** Association between the ability to actively interact with health professionals (Subscale 6 – *Health Literacy Questionnaire*) and sociodemographic and clinical variables of adults with Diabetes *Mellitus* Type 2 – Metropolitan Region of Curitiba, PR, Brazil, 2024.

Variables	n	Mean ± SD	p-value
Age (years)			
40–59	106	3.65 ± 0.88	0.684*
60–65	63	3.70 ± 0.89	
(total n)	169		
Sex			
Female	98	3.56 ± 0.93	0.057*
Male	71	3.82 ± 0.80	
(total n)	169		
Education (years)			
Not literate	10	3.24 ± 0.68	0.443**
≤ 5	63	3.71 ± 0.89	
6–9	33	3.70 ± 1.01	
10–12	41	3.77 ± 0.78	
> 12	22	3.51 ± 0.91	
(total n)	169		
HbA1c (%)			
< 7	35	3.85 ± 0.87	0.231*
≥ 7	130	3.64 ± 0.88	
(total n)	165		
Fasting Blood Glucose (mg/dL)			
≤ 130	60	3.88 ± 0.81	**0.033**
> 130	105	3.58 ± 0.90	
(total n)	165		

*Student's t-test for independent samples or non-parametric Mann-Whitney test, p < 0.05;

**One-way ANOVA with a non-parametric factor or Kruskal-Wallis test, p < 0.05.

The mean score for subscale 9, i.e. understanding health information and knowing what to do, was 3.20 ± 0.91 (95% CI: 3.10–3.40), which shows the association between the level of education and HL skills (*p* = <0.001), with illiterate participants with ≤ 5 years of formal education obtaining lower means and SD on the subscale (Table 3).

**Table 3:** Association between understanding health information and knowing what to do (Subscale 9 – from *Health Literacy Questionnaire*) with the sociodemographic and clinical variables of adults with Diabetes *Mellitus* Type 2 – Metropolitan Region of Curitiba, PR, Brazil, 2024.

Variables	n	Mean ± SD	p-value
Age (years)			
40–59	106	3.24 ± 0.93	0.445*
60–65	63	3.13 ± 0.88	
(total n)	169		
Sex			
Female	98	3.19 ± 0.99	0.903*
Male	71	3.21 ± 0.78	
(total n)	169		
Education (years)			
Not literate	10	1.92 ± 0.30	<0.001**
≤ 5	63	2.88 ± 0.80	
6–9	33	3.41 ± 0.82	
10–12	41	3.57 ± 0.85	
> 12	22	3.69 ± 0.75	
(total n)	169		
HbA1c (%)			
< 7	35	3.41 ± 0.80	0.134*
≥ 7	130	3.15 ± 0.94	
(total n)	165		
Fasting Blood Glucose (mg/dL)			
≤ 130	60	3.33 ± 0.92	0.201*
> 130	105	3.14 ± 0.91	
(total n)	165		

*Student's t-test for independent samples or non-parametric Mann-Whitney test, p < 0.05;

**One-way ANOVA with a non-parametric factor or Kruskal-Wallis test, p < 0.05.

Table 4 shows the weak correlation between education and family income with subscale 9 of the HLQ-Br (*r* = 0.50 and 0.35, respectively) and significant value with HbA1c (*p* = 0.031).

**Table 4:** Correlation of subscales 6 and 9 of the *Health Literacy Questionnaire* with the sociodemographic and clinical variables of adults with Type 2 Diabetes Mellitus – Metropolitan Region of Curitiba, PR, Brazil, 2024.

Variables	N	r	p-value*
Subscale 6			
Age	169	-0.001	0.985
Level of Education	169	0.04	0.600
Family income	168	0.12	0.123
Diagnostic time	169	0.01	0.907
HbA1c	165	-0.07	0.364
Fasting blood glucose	165	-0.09	0.230
Subscale 9			
Age	169	-0.10	0.184
Level of Education	169	**0.50**	**< 0.001**
Family income	168	**0.35**	**< 0.001**
Diagnostic time	169	-0.09	0.243
HbA1c	165	-0.17	**0.031**
Fasting blood glucose	165	-0.05	0.518

*Spearman's Correlation Coefficients, p < 0.05.

When analyzing Cronbach’s alpha coefficient of the subscales related to the ability to actively interact with health professionals and understand health information and know what to do, values of 0.82 and 0.74 were obtained, respectively, which are considered satisfactory.

## DISCUSSION

The results of this study reveal a significant relationship between sociodemographic and clinical variables and the two subscales of the HLQ-Br. It was found that men and those with lower fasting blood glucose levels had greater HL skills in terms of interaction with health professionals, allowing inferring that health-related guidelines enabled the understanding of the importance of the therapeutic plan and the adoption of healthy lifestyle habits in terms of better monitoring and effectiveness of blood glucose levels.

A similar result was found in research carried out in Africa, which showed a significant relationship between subscale 6 of the HLQ and male sex (*p* = 0.006)^(15)^. On the other hand, British research found no association between such variables^(8)^. Study conducted in Rio de Janeiro, Brazil, with 107 participants followed in a DM outpatient clinic, which used the *European Health Literacy Survey*, through the simple ordinal regression model, showed that male participants were three times more likely to have an excellent HL level (*p* = 0.03)^(16)^. In this regard, it is believed that given men’s experiences of chronic illness, there is a mobilization in the search for knowledge and social support with health services and professionals, for the development of skills that promote physical and mental well-being^(17)^.

It is to be noted that the National Policy for Comprehensive Attention to Men’s Health (*PNAISH*), of the Brazilian Ministry of Health, published in 2009, emphasizes in its objectives the promotion of changes in paradigms related to the perception of the male population about health care, as well as the qualification of professionals in the basic network to meet the demands of this population^(18)^. However, research indicated that ongoing education and support for the management of care for men occurred in an incipient and fragmented manner, due to the lack of training and theoretical-practical basis for professionals^(19)^. Divergent results were found in a study that showed that men were unaware of health policies and felt dissatisfied with the care they received, due to the lack of professional support, material resources and infrastructure, and stated that women received more attention than they did in PHC^(20)^.

The association between the ability to interact with health professionals and glycemic levels showed that participants with fasting glycemia ≤ 130 mg/dL had greater HL skills in this regard, allowing inferring that they felt safer to expose their experiences and health conditions, without fear of impositions or judgments from professionals. Thus, an Australian study, which aimed to explore what participants with DM would like their health professionals to understand about living with the disease, resulted in perceptions that indicated a lack of person-centered care, and that professionals’ judgments, assumptions, and negative perspectives did not help them manage the disease^(21)^.

Health professionals are jointly responsible for developing self-management skills, through health education and support actions, which are aimed at basic information for managing the disease and care, strengthening changes in lifestyle habits and psychosocial support, recognizing the emotional burden of living with and managing DM. Educational actions provide knowledge, confidence, and improvement of skills to manage care, with the aim of equipping the person to make decisions and increase problem-solving ability^(22)^.

In this sense, the importance of assessing HL in people with T2DM is reinforced, with the intention of recognizing the search and sources of information, interpretation, and judgment regarding the care and needs demanded by the disease. Therefore, cultural differences and social determinants of health must be acknowledged to promote equitable and person-centered care^(22)^, increasing HL skills of the population with T2DM.

Regarding the association of education with the skills of understanding health information and knowing what to do, participants with less time of formal education had the lowest HL averages, when compared to those who had 12 or more years of education. It can be deduced that the lack and/or incompleteness of participants’ writing and reading skills compromise obtainment and interpretation of health information, hindering judgment in the face of adversities imposed by illness. These factors have a negative impact on the life of people with T2DM, as a lack of a global understanding of the disease can lead to the risk of complications, a worsening of quality of life, and an increase in the number of hospitalizations and costly expenses for the individual and the health system^(23)^.

It is worth noting that more than half of the population in this study (n = 106) had less than 10 years of schooling, a result similar to the research carried out in Burkina Faso, in West Africa, which obtained a higher concentration of participants with primary education (n = 175; 77.7%), this variable being associated with low ability to understand health information and know what to do, through the application of the HLQ (*p* = 0.004)^(15)^.

Another study, conducted in Spain with 252 participants diagnosed with cardiovascular diseases, found an association between subscale 9 of the HLQ and the level of education (*p =* < 0.001) and social class (*p =* < 0.001), with the largest concentration of participants having a basic educational level (50.0%) and being from the middle social class (81.7%)^(24)^. Although this study did not obtain statistical significance in the association between subscale 9 of the HLQ and family income, a weak correlation was found between the ability to understand health information and knowing what to do with the variables education, family income, and HbA1c.

It is noted that people with low levels of education tend to have greater difficulties in entering the formal job market, leading to precarious employment, especially with regard to remuneration, which consequently places them in a condition of poverty^(25)^. In this regard, when considering the low levels of education and income, and high glycemic rates of the participants in this study, we reflect on knowing what to do, due to the financial barriers to accessing dietary products, which help in glycemic control, such as foods that are sources of fiber, including fruit and vegetables, dairy products, and lean meat^(26)^. It can be inferred that another obstacle, related to the level of education, concerns the understanding of guidelines and the adequate interpretation of food labels for healthier choices.

In a cross-sectional study with 33 participants with T2DM, a weak correlation was found between fasting blood glucose levels and family income (*r* = 0.1946) and a moderate correlation with knowledge about the disease (*r* = 0.4260), indicating that the higher the family income, the lower the glycemic values and the better the understanding of DM^(27)^. Given these conditions, PHC professionals must adapt their forms of communication and language, and health services and systems must be improved to offer support and tools to minimize discrepancies related to HL^(28)^, especially people with low levels of education and income.

In this regard, health education actions, based on HL, are strategies to allow the development of skills that promote knowledge and self-efficacy. The effects of these strategies were verified in a randomized clinical trial with 107 participants with DM, of which 54 belonged to the intervention group, who received counseling by telephone. After the six- month period, participants in the intervention group with low levels of HL had significant improvement in self-efficacy (*p* = 0.000), as well as those with high levels (*p* = 0.016)^(29)^. Thus, it can be inferred that the use of educational actions aimed at HL can help to level interpretative and decision- making skills, especially to understand the complexity related to DM management.

The applicability of subscales 6 and 9 of the HLQ-Br for the adult population with T2DM showed good consistency and the values for the Cronbach’s alpha coefficient were similar to those obtained in the validation of the instrument for Brazilian Portuguese, being 0.78 for the subscale ability to interact with health professionals and 0.76 for understanding health information and knowing what to do^(13)^.

This study had some limitations that should be considered when interpreting the results. First, the presence of companions during the application of the questionnaire, which may have affected the interpretation of the questions, especially when carried out in the form of an interview. The sample, although representative, may not adequately reflect the diversity of the population of adults with T2DM, limiting the generalization of the findings to broader contexts. It should be considered that studies on HL in DM have significant methodological limitations, due to the cross-sectional typology and the lack of standardization in measurement tools, which makes comparison between studies difficult^(23)^.

External factors, such as the socioeconomic situation, policies, and health programs in force in the country, may have influenced the levels of HL, such as the funding model of the Brazilian Public Health System (SUS), the *Previne Brasil* Program, which among its criteria had payment for performance with the objective of expanding services and periodically monitoring users with DM. However, in 2024, the program underwent significant changes with the implementation of new financing components^(30)^. Such considerations are important in the interpretation of results and for future research on HL among adults with T2DM.

## CONCLUSION

It was concluded that there was a relationship between the ability to interact with health professionals and the male sex, showing that men had greater criticality for health care, in the search for information and questions to professionals, as well as those who had low fasting blood glucose levels. A weak correlation was found between understanding health information and knowing what to do, family income, education and HbA1c, results that can contribute to the development of educational actions aimed at strengthening HL.

It is believed that the implementation of continuing education programs for PHC professionals, with a focus on adapting simple language, can optimize interaction and understanding of information for users of health services with T2DM, especially women, those with low levels of education and with fasting blood glucose levels >130mg/dL, to reduce disease-related complications and empower people to self-manage their care. Furthermore, it is essential that public policies are developed to address HL inequalities, especially in populations with lower education and income.

Future research is encouraged to explore specific interventions that aim to improve HL skills and assess their impact on the health conditions and quality of life of the population. These initiatives are essential to promote more equitable and person- centered care, contributing to the mitigation of complications associated with T2DM, and to strengthening autonomy and health promotion.

## References

[1] Nutbeam D, Lloyd JE. (2021). Understanding and responding to health literacy as a social determinant of health.. Annu Rev Public Health.

[2] Nutbeam D. (1998). Health promotion glossary. Health Promot Int.

[3] Sørensen K, Brand H (2014). Health literacy lost in translations? Introducing the European health literacy glossary. Health Promot Int.

[4] Paes RG, Mantovani MF, Costa MC, Pereira ACL, Kalinke LP, Moreira RC (2022). Effects of educational intervention on health literacy and knowledge about diabetes: a quasi-experimental study. Esc Anna Nery.

[5] Brasil. (2021). Plano de ações estratégicas para o enfrentamento das doenças crônicas e agravos não transmissíveis no Brasil 2021-2030 [Internet]..

[6] Rodacki M, Teles M, Gabbay M, Lamounier R, Montenegro R, Bertoluci M. (2023). Classificação do diabetes..

[7] International Diabetes Federation. (2021). IDF diabetes atlas [Internet]..

[8] Simpson RM, Knowles E, O’Cathain A (2020). Health literacy levels of British adults: a cross-sectional survey using two domains of the Health Literacy Questionnaire (HLQ). BMC Public Health.

[9] Witiski M, Makuch DMV, Rozin L, Matia G (2019). Communication barriers: perception of a healthcare team. Ciênc Cuid Saúde.

[10] Tefera YG, Gebresillassie BM, Emiru YK, Yilma R, Hafiz F, Akalu H (2020). Health literacy and its association with glycemic control among adults with type 2 diabetes at a university hospital in Ethiopia. PLoS One.

[11] Cuschieri S. (2019). The STROBE guidelines. Saudi J Anaesth.

[12] Instituto Brasileiro de Geografia e Estatística. (2022). Cidades e Estados: Colombo [Internet]..

[13] Moraes KL, Brasil VV, Mialhe FL, Sampaio HAC, Sousa ALL, Canhestro MR (2021). Validation of the Health Literacy Questionnaire (HLQ) to brazilian Portuguese. Acta Paul Enferm.

[14] Osborne RH, Batterham RW, Elsworth GR, Hawkins M, Buchbinder R (2013). The Health Literacy Questionnaire (HLQ): development and initial validation of nine scales to comprehensively measure health literacy. BMC Public Health.

[15] Nacanabo R, Debussche X, Rouamba M, Kamouni P, Mancini J, Kouanda S (2021). Health literacy and health-related quality of life in type 2 diabetes: a cross-sectional study in Burkina Faso. Diabetes Epidemiol Manage.

[16] Pavão ALB, Werneck GL, Saboga-Nunes L, Sousa RA (2021). Avaliação da literacia para a saúde de pacientes portadores de diabetes acompanhados em um ambulatório público. Cad Saude Publica.

[17] Sousa AR, Silva AF, Estrela FM, Bonfim HP, Sousa TJ, Conceição LN (2021). Health care praxiology men living with diabetes and hypertension. REVISA.

[18] Brasil (2009). Ministério da Saúde. Portaria nº 1.944, de 27 de agosto de 2009 [Internet]..

[19] Berbel CMN, Quaglio Chirelli M (2020). Reflexões do cuidado na saúde do homem na atenção básica. Rev Bras Promoc Saúde.

[20] Silva CD, Souza JR, Silva NS, Almeida SP, Torres LM (2022). Saúde do homem na atenção básica: fatores que influenciam a busca pelo atendimento. Rev Cienc Plural.

[21] Litterbach E, Holmes-Truscott E, Pouwer F, Speight J, Hendrieckx C (2020). ‘I wish my health professionals understood that it’s not just all about your HbA1c!’: qualitative responses from the second Diabetes MILES – Australia (MILES-2) study. Diabet Med.

[22] Davis J, Fischl AH, Beck J, Browning L, Carter A, Condon JE (2022). 2022 National Standards for Diabetes Self-Management Education and Support. Diabetes Spectr.

[23] Visscher BB, Steunenberg B, Heijmans M, Hofstede JM, Devillé W, van der Heide I (2018). Evidence on the effectiveness of health literacy interventions in the EU: a systematic review. BMC Public Health.

[24] Cabellos-García AC, Castro-Sánchez E, Martínez-Sabater A, Díaz-Herrera MA, Ocaña-Ortiz A, Juárez-Vela R (2020). Relationship between determinants of health, equity, and dimensions of health literacy in patients with cardiovascular disease. Int J Environ Res Public Health.

[25] Couto ACL, Silva C. Pobreza (2022). escolaridade e formas de inserção no mercado de trabalho. Uma análise para o Brasil nos anos de 2012 e 2019. Rev Orb Lat..

[26] Ramos S, Campos LF, Baptista DR, Strufaldi M, Gomes DL, Guimarães DB (2023). Terapia Nutricional no Pré-Diabetes e no Diabetes Mellitus Tipo 2..

[27] Paes RG, Mantovani MF, Boller C, Costa MC, Piccinin VP, Spinelli SMC (2023). Knowledge about diabetes and health literacy: analysis by item response theory. Rev Enf UFPE on line.

[28] Lima RIM, Parente MA, Ferreira TISP, Coelho AAS, Loureiro EVS, Barbosa TM (2022). Functional health literacy in users of Family health units from Altamira (state of Pará, Brazil). Rev Bras Med Fam Comunidade.

[29] Ag?rali H, Akyar I (2022). The effect of health literacy-based, health belief-constructed education on glycated hemoglobin (HbA1c) in people with type 2 diabetes: a randomized controlled study. Prim Care Diabetes.

[30] Brasil (2024). Ministério da Saúde. Portaria GM/MS nº 3.493, de 10 de abril de 2024. Altera a Portaria de Consolidação de GM/MS nº 6, de setembro de 2017, para instituir nova metodologia de cofinanciamento Federal do Piso de Atenção Primária à Saúde do SUS [Internet]..

