# Supplementary material for: Health literacy of adults from primary care with type 2 diabetes mellitus: a cross-sectional study
Source: Rev Esc Enferm USP. 2025 May 16;59:e20240338. doi: 10.1590/1980-220X-REEUSP-2024-0338en (PMC12090903; doi:10.1590/1980-220X-REEUSP-2024-0338en)
Supplement: Translation

# Translation: Letramento em saúde de adultos da atenção primária com diabetes *mellitus* tipo 2: estudo seccional

**DOI:** 10.1590/1980-220X-REEUSP-2024-0338pt

**Authors:** Robson Giovani Paes, Isabella Bueno Fusculim, Luciane Lachouski, Ingrid Marcela Pinto Gariba de Andrade, Shirley Boller, Maria de Fátima Mantovani

**Affiliations:** 1 Universidade Federal do Paraná, Curitiba, PR, Brasil.

**Keywords:** Enfermagem, Diabetes Mellitus, Letramento em Saúde, Atenção Primária à Saúde

## Abstract

**Objetivo:**

Relacionar o letramento em saúde de adultos com diabetes *mellitus* tipo 2 da atenção primária à saúde com as variáveis sociodemográficas e clínicas.

**Método:**

Estudo descritivo analítico seccional, com 169 participantes de 18 a 65 anos com diabetes *mellitus* tipo 2, de seis unidades da atenção primária da Região Metropolitana de Curitiba, Paraná, Brasil. A coleta de dados ocorreu de julho de 2023 a março de 2024, e utilizou-se questionário sociodemográfico e clínico, e as subescalas seis e nove do *Health Literacy Questionnaire* versão brasileira. Analisaram-se os dados descritivamente e pelos testes T de Student, Mann-Whitney, Anova, Kruskal-Wallis e Spearman.

**Resultados:**

Dos 169 participantes, a média de idade foi de 56,5 ± 6,2 anos. Verificou-se associação significativa entre a capacidade de interagir com os profissionais de saúde e o sexo masculino (*p* = 0,057), glicemia de jejum ≤130 mg/dL (*p* = 0,033), e entre a escolaridade e compreender as informações sobre saúde e saber o que fazer (*p* = <0,001). Encontrou-se fraca correlação entre compreender as informações sobre saúde e saber o que fazer e a escolaridade e a renda familiar (*r* = 0,50 e 0,35, respectivamente) e valor significativo com a hemoglobina glicada (*p* = 0,031).

**Conclusão:**

Verificou-se relação do letramento em saúde com o sexo masculino, escolaridade, renda familiar e valores glicêmicos. Esses resultados podem contribuir para o desenvolvimento de ações educativas voltadas ao fortalecimento do letramento em saúde.

## INTRODUÇÃO

O letramento em saúde (LS) é um potencial determinante social de saúde^(1)^ que expressa o grau de capacidade que o indivíduo dispõe para receber, processar e compreender as informações e serviços básicos, necessários para tomar as decisões adequadas em relação a seu estado de saúde^(1,2)^. É definido como o conhecimento, motivação e competências para acessar, avaliar e aplicar informações no campo da prevenção de doenças, de cuidados e promoção à saúde, a fim de melhorar ou manter a qualidade de vida e a autonomia decisória^(2)^.

O LS também pode ser conceituado como um conjunto de habilidades individuais e sociais, pela partilha informacional entre os profissionais, usuários e sistemas, no fortalecimento do oportuno julgamento para a tomada de decisão em saúde^(3)^. Ao analisar os níveis de LS, é possível identificar o grau de envolvimento do indivíduo com o processo de saúde-doença, o seu reconhecimento dos fatores de risco, o acesso às informações e aos serviços, na possibilidade de criar ações para participação ativa nos cuidados relativos à saúde^(4)^.

A análise do LS está em concordância com as medidas preventivas e de vigilância às doenças e agravos crônicos não transmissíveis, apresentadas no plano de ações estratégicas do Ministério da Saúde (MS) brasileiro, que preconiza a importância do conhecimento das pessoas sobre os fatores de risco, o acesso a bens e serviços públicos, e a garantia de direitos de informações, que permitam escolhas favoráveis à saúde^(5)^.

Ao considerar as doenças crônicas não transmissíveis, o diabetes *mellitus* (DM) destaca-se como um importante problema de saúde pública, em decorrência de sua prevalência e complicações^(6)^. O diabetes *mellitus* tipo 2 (DM2) representa cerca de 90–95% dos casos da doença e têm como fatores desencadeantes o histórico familiar, a dificuldade de controle do peso e hábitos de vida sedentários, respondendo por altas taxas de complicações micro e macrovasculares, como acidente vascular cerebral, infarto agudo do miocárdio, amputações, retinopatia, neuropatia e outros problemas^(6)^.

Em 2021, o Brasil encontrava-se na sexta colocação no *ranking* mundial da doença, com aproximadamente 15 milhões de pessoas com DM na faixa etária de 20 a 79 anos, com projeção de aumento de 7,5 milhões de novos casos para o ano de 2045^(7)^. Esta projeção torna-se preocupante na vigência de complicações tipicamente comuns ao DM2, as quais podem ser relacionadas aos baixos níveis de LS e ao desconhecimento da doença, que podem interferir diretamente na obtenção e na compreensão das informações sobre saúde^(4)^.

Esses aspectos foram apontados em um estudo anterior realizado com 33 adultos diagnosticados com DM2, cadastrados em unidade de saúde da região sul do Brasil, aos quais foram aplicados os instrumentos *Spoken Knowledge in Low Literacy Patients with Diabetes* (SKILLD) e *Eight-Item Health Literacy Assessment Tool* (HLAT-8) para analisar o conhecimento sobre a doença e o LS. Após a intervenção educativa de enfermagem, verificou-se aumento do conhecimento sobre a DM (*p* = 0,001) correlacionado ao LS (*p* = 0,001)^(4)^, podendo-se inferir que a interação do profissional e as ações centradas na pessoa auxiliaram os participantes na busca e no entendimento de informações e serviços de saúde.

Nesse sentido, buscou-se entender a capacidade de interagir com os profissionais de saúde e de compreender as informações de saúde e de saber o que fazer, dos adultos com DM2, baseando-se em um estudo britânico com 2.309 participantes, o qual relacionou as subescalas seis e nove do *Health Literacy Questionnaire* (HLQ) relacionadas às habilidades de interação com os profissionais e compreensão das informações de saúde, com as variáveis sociodemográficas e clínicas. Os resultados mostraram que 19,4% dos participantes apresentavam dificuldades para ler e compreender as informações escritas de saúde e 23,2% de discutir suas preocupações com os profissionais de saúde devido à baixa condição social e de escolaridade. Cabe destacar que os participantes tinham pelo menos uma doença crônica^(8)^.

Assim, torna-se imprescindível identificar as barreiras de comunicação, sejam elas pela linguagem tecnicamente complexa, utilizada pelo profissional de saúde, ou pela incorreta ou incompleta informação captada pelo interlocutor, fatores esses que podem interferir no cumprimento das intervenções de saúde, no agravamento do quadro clínico^(9)^ e no desenvolvimento de habilidades de LS. Desvela-se que pessoas com DM2 com níveis elevados de LS tendem a melhor adesão ao tratamento medicamentoso e a mudanças no estilo de vida, com desfecho no controle glicêmico e na mitigação das complicações associadas à doença^(10)^.

Nesse sentido, com a finalidade de contribuir para o cuidado voltado para a integralidade da assistência às pessoas com DM2 no contexto da Atenção Primária à Saúde (APS), tendo como base o grau de LS, este estudo objetivou relacionar o letramento em saúde de adultos com diabetes *mellitus* tipo 2 da atenção primária à saúde com as variáveis sociodemográficas e clínicas.

## MÉTODO

### Desenho do Estudo

Estudo quantitativo, descritivo, analítico, seccional que seguiu as recomendações da *Strengthening the Reporting of Observational studies in Epidemiology* (Strobe)^(11)^.

### Local, População e Critérios de Seleção

O estudo foi conduzido em seis unidades da APS localizadas na Região Metropolitana de Curitiba, Paraná, Brasil. O município estava entre as dez cidades mais populosas do Estado, com aproximadamente 232.212 habitantes e com o Índice de Desenvolvimento Humano Municipal (IDHM) de 0,733, considerado similar ao do país. Sua distribuição territorial era de 197,580 km^2^ com densidade demográfica de 1.175,28 habitantes/km^2(12)^.

Na esfera da saúde, o município contava com 25 unidades da APS, organizadas em três distritos sanitários, com cerca de 11.278 usuários diagnosticados com DM e cadastrados no Programa de hipertensão e DM do Ministério da Saúde (Hiperdia).

Os critérios de elegibilidade foram usuários dos serviços de saúde com idade entre 18 e 65 anos, cadastrados no Programa Hiperdia e com diagnóstico confirmado de DM2. Foram excluídos os usuários com comorbidades cognitivas e/ou de comunicação, identificadas pela descrição no prontuário eletrônico.

### Definição da Amostra e Recrutamento

O estudo integra um projeto maior que utilizou uma amostragem probabilística de base populacional por conglomerados. Essa amostragem foi planejada com base no número estimado de habitantes do município, considerando uma taxa de prevalência nacional de DM de 10,5% (intervalo de confiança [IC] de 95%: 9,4% a 11,6%), um erro aceitável de 5% e uma amostra heterogênea (50/50). O número de participantes por conglomerado foi definido proporcionalmente ao total de usuários cadastrados com DM de cada unidade, e neste subprojeto, os dados referem-se a 25% (seis unidades da APS), sendo duas de cada distrito sanitário.

A seleção dos participantes foi realizada por meio de um aplicativo de sorteio on-line, utilizando os nomes de todos os usuários constantes nas listas do Programa Hiperdia, fornecidas pelas coordenações das unidades da APS. Esse procedimento garantiu a aleatoriedade da amostra, permitindo o acesso posterior aos prontuários para identificar e definir os usuários elegíveis. As listas foram organizadas com a inclusão de suplentes, de forma a substituir voluntários em caso de recusas ou contatos inexistentes.

O recrutamento dos usuários sorteados foi realizado simultaneamente à coleta de dados, por meio de ligações telefônicas e/ou mensagens de texto padrão enviadas pelo aplicativo WhatsApp®️. Foram feitas até três tentativas de contato, em dias e horários diferentes, para convidar os usuários a participar do estudo. Nos casos de aceite, agendava-se uma consulta de enfermagem, a ser realizada na unidade de saúde ou no domicílio, conforme a disponibilidade e preferência do participante. Ao todo, foram contatados 596 usuários: 381 não atenderam às ligações ou não responderam às mensagens, 46 recusaram participar e 169 compuseram a amostra final.

### Instrumentos de Coleta de Dados

Para a coleta de dados foram utilizados dois instrumentos: um questionário contendo variáveis sociodemográficas e clínicas e o HLQ versão brasileira (HLQ-Br). O questionário continha as variáveis sociodemográficas (idade, sexo, escolaridade, estado conjugal e renda familiar) e clínicas (tempo de diagnóstico de DM2, exames de hemoglobina glicada (HbA1c) e glicemia de jejum).

O HLQ-Br é um instrumento multidimensional desenvolvido na Austrália, que pode ser autoadministrado ou aplicado na forma de entrevista. Contém 44 questões distribuídas em nove subescalas: 1 – Compreensão e apoio dos profissionais de saúde; 2 – Informações suficientes para cuidar da saúde; 3 – Cuidado ativo da saúde; 4 – Suporte social para saúde; 5 – Avaliação das informações em saúde; 6 – Capacidade de interagir ativamente com os profissionais de saúde; 7 – Navegar no sistema de saúde; 8 – Capacidade de encontrar boas informações sobre saúde; e 9 – Compreender as informações sobre saúde e saber o que fazer. Foi validado para o português do Brasil^(13,14)^.

O HLQ-Br é uma ferramenta que auxilia na identificação das condições de LS. Para este estudo, foram selecionadas as subescalas 6 e 9, que abordam questões relacionadas à comunicação com profissionais de saúde e à compreensão e seguimento de instruções de saúde. As respostas destas subescalas seguem o formato Likert de cinco pontos, que varia de “sempre difícil (1)” a “sempre fácil (5)”. A pontuação do HLQ foi calculada com base na média dos itens de cada subescala.

### Coleta de Dados

A coleta de dados foi realizada durante a consulta de enfermagem, com duração média de 50 minutos, conduzida pelo pesquisador principal e 10 auxiliares de pesquisa, que receberam treinamento prévio de cinco horas, mediante curso teórico-prático que envolviam os temas de LS e DM.

Aplicou-se o questionário sociodemográfico e clínico com as variáveis de idade, sexo, escolaridade, estado conjugal, renda familiar e tempo de diagnóstico de DM2, que foram obtidas por autodeclaração. Salienta-se que um participante não soube ou não quis informar a renda familiar mensal.

Os valores dos exames de HbA1c e da glicemia de jejum foram obtidos do prontuário do participante, sendo válidos até um ano antes do estudo. Para os casos em que as variáveis bioquímicas estavam fora do prazo estabelecido, solicitou-se a realização dos exames nos laboratórios credenciados à secretaria de saúde do município, por intermédio do Programa Previne Brasil do MS. Ressalta-se que a solicitação para a coleta de exames foi entregue para 25 participantes, porém quatro não realizaram, reduzindo o número da amostra para esta variável.

Posteriormente foram aplicadas as duas subescalas do HLQ-Br, em aproximadamente 20 minutos. Para os participantes com baixa visão e dificuldades de leitura, realizou-se a aplicação do instrumento na forma de entrevista.

### Análise e Tratamento dos Dados

Os dados foram inseridos em planilhas do *Microsoft Excel* ^®^ por dois pesquisadores de forma independente, sendo realizado confronto de ambas planilhas pelo profissional de estatística, a fim de averiguar quaisquer inconsistências, e os dados foram analisados com auxílio do programa computacional IBM^®^ *Statistical Package for Social Sciences* (SPSS) v.29.0.0. A análise das variáveis descritivas quantitativas (idade, escolaridade, renda familiar, tempo de diagnóstico, valores de HbA1c e glicemia de jejum) foi apresentada por média e desvio padrão (DP).

As variáveis categóricas foram descritas por frequência absoluta e percentual, sendo idade (45–59; 60–65 anos), sexo (masculino/feminino), estado conjugal (solteiro(a)/casado(a)/separado(a) ou divorciado(a)/viúvo(a)), escolaridade (não alfabetizado(a); ≤ 5; 6–9; 10–12 e > 12 anos), renda familiar (< 1.320; 1.320–3.960; > 3.960 reais por mês), tempo de diagnóstico (< 1; 1–5; 6–10 e > 10 anos), HbA1c (< 7 e ≥ 7%) e glicemia de jejum (≤ 130 e > 130 mg/dL).

Para analisar a associação das subescalas do questionário HLQ-Br com variáveis sociodemográficas e clínicas categóricas com duas classificações, foi usado o teste T de Student para amostras independentes ou o teste não-paramétrico de Mann-Whitney. Variáveis com mais de duas classificações foram analisadas pelo modelo de análise da variância (Anova) com um fator ou o teste não-paramétrico de Kruskal-Wallis. Para a análise de correlação entre as subescalas e as variáveis quantitativas, foram estimados Coeficientes de Correlação de Spearman (*r*). Interpretaram-se os valores de correlação como excelente (*r =* > 0,90), bom (*r =* 0,75 a 0,90), moderado (*r* = 0,50 a 0,74) e fraco (*r =* < 0,50). Os valores de p < 0,05 indicaram significância estatística.

Os resultados das subescalas 6 e 9 do HLQ-Br foram apresentados em medida pontual e estimada, com IC de 95%. Testou-se a confiabilidade das subescalas para a população adulta com DM2 pelo Coeficiente de alfa de Cronbach (α), sendo considerado o valor mínimo de 0,70.

### Aspectos Éticos

O estudo foi aprovado pelo Comitê de Ética em Pesquisa com Seres Humanos de Universidade Pública do Estado do Paraná sob o parecer consubstanciado nº. 6.138.731 e CAAE 68471723.6.0000.0102. Para o desenvolvimento da pesquisa obedeceram-se às exigências do Conselho Nacional de Saúde (CNS) dispostas na Resolução nº. 466/2012.

O HLQ foi elaborado pela Universidade Tecnológica de Swinburne, na Austrália, a qual detém os direitos de propriedade intelectual do material. Para a sua aplicabilidade neste estudo, obteve-se a licença pelo código L23009IS, e seguiram-se as recomendações dos autores para a apresentação das questões do instrumento^(14)^.

## RESULTADOS

Dos 169 (100%) participantes com DM2, 58,0% (n = 98) eram mulheres, com idade entre 40 e 65 anos (56,5 ± 6,2), renda familiar média de R$ 4.032 ± 3.021 e 37,3% (n = 63), com escolaridade igual ou menor a cinco anos e 75,1% (n = 127) eram casados(as) e/ou união consensual. No que tange às variáveis clínicas, 78,9% (n = 130) apresentaram valores de Hb1Ac ≥ 7%, 63,6% (n = 105) com glicemia de jejum > 130 mg/dL e 46,7% (n = 79) e haviam recebido o diagnóstico de DM2 há mais de 10 anos (Tabela 1).

**Tabela 1:** Caracterização sociodemográfica e clínica dos adultos com Diabetes *Mellitus* Tipo 2 – Região Metropolitana de Curitiba, PR, Brasil, 2024.

Variáveis	n (%)	Média ± dp
Idade (anos)		
40–59	106 (62,7)	56,5 ± 6,2
60–65	63 (37,3)	
(n total)	169 (100)	
Sexo		
Feminino	98 (58,0)	
Masculino	71 (42,0)	
(n total)	169 (100)	
Estado conjugal		
Solteiro(a)	15 (8,9)	
Casado(a)/união consensual	127 (75,1)	
Separado(a)/divorciado(a)	19 (11,2)	
Viúvo(a)	8 (4,7)	
(n total)	169 (100)	
Escolaridade (anos)		
Não alfabetizado(a)	10 (5,9)	7,6 ± 4,7
≤ 5	63 (37,3)	
6–9	33 (19,5)	
10–12	41 (24,3)	
> 12	22 (13,0)	
(n total)	169 (100)	
Renda familiar (reais)*		
< 1,320	17 (10,1)	4,032 ± 3,021
1.320–3.960	78 (46,4)	
> 3,960	73 (43,5)	
(n total)	168 (100)	
Tempo de diagnóstico (anos)		
< 1	1 (0,6)	11,89 ± 8,6
1–5	48 (28,4)	
6–10	41 (24,3)	
> 10	79 (46,7)	
(n total)	169 (100)	
HbA1c (%)		
< 7	35 (21,2)	8,7 ± 2,1
≥ 7	130 (78,8)	
(n total)	165 (100)	
Glicemia de Jejum (mg/dL)		
≤ 130	60 (36,4)	173,6 ± 81,7
> 130	105 (63,3)	
(n total)	165 (100)	

*Referente ao salário-mínimo brasileiro em julho de 2023.

A média de LS na subescala 6, ou seja, capacidade de interagir ativamente com os profissionais de saúde foi de 3,67 ± 0,88 (IC 95%: 3,53–3,80) e apresentou associação significativa com o sexo masculino (p = 0,057) e àqueles que apresentavam valores de glicemia de jejum ≤ 130 mg/dL (p = 0,033) (Tabela 2).

**Tabela 2:** Associação entre capacidade de interagir ativamente com os profissionais de saúde (Subescala 6 – do *Health Literacy Questionnaire*) com as variáveis sociodemográficas e clínicas dos adultos com Diabetes *Mellitus* Tipo 2 - Região Metropolitana de Curitiba, PR, Brasil, 2024.

Variáveis	n	Média ± dp	p-valor
Idade (anos)			
40–59	106	3,65 ± 0,88	0,684*
60–65	63	3,70 ± 0,89	
(n total)	169		
Sexo			
Feminino	98	3,56 ± 0,93	0,057*
Masculino	71	3,82 ± 0,80	
(n total)	169		
Escolaridade (anos)			
Não alfabetizado(a)	10	3,24 ± 0,68	0,443**
≤ 5	63	3,71 ± 0,89	
6–9	33	3,70 ± 1,01	
10–12	41	3,77 ± 0,78
> 12	22	3,51 ± 0,91
(n total)	169	
HbA1c (%)			
< 7	35	3,85 ± 0,87	0,231*
≥ 7	130	3,64 ± 0,88	
(n total)	165		
Glicemia de Jejum (mg/dL)			
≤ 130	60	3,88 ± 0,81	0,033*
> 130	105	3,58 ± 0,90	
(n total)	165		

*Teste t de Student para amostras independentes ou teste não-paramétrico de Mann-Whitney, p < 0,05;

**ANOVA com um fator ou teste não-paramétrico de Kruskal-Wallis, p < 0,05.

A média da subescala 9, ou seja, compreender as informações sobre saúde e saber o que fazer, foi de 3,20 ± 0,91 (IC 95%: 3,10–3,40), o que mostra a associação entre o nível de escolaridade e as habilidades de LS (*p* = <0,001), sendo que os participantes não alfabetizados e com tempo de estudo formal ≤ 5 anos obtiveram menores médias e DP na subescala (Tabela 3).

**Tabela 3:** Associação entre compreender as informações sobre saúde e saber o que fazer (Subescala 9 – do *Health Literacy Questionnaire*) com as variáveis sociodemográficas e clínicas dos adultos com Diabetes *Mellitus* Tipo 2 – Região Metropolitana de Curitiba, PR, Brasil, 2024.

Variáveis	n	Média ± dp	p-valor
Idade (anos)			
40–59	106	3,24 ± 0,93	0,445*
60–65	63	3,13 ± 0,88	
(n total)	169		
Sexo			
Feminino	98	3,19 ± 0,99	0,903*
Masculino	71	3,21 ± 0,78	
(n total)	169		
Escolaridade (anos)			
Não alfabetizado	10	1,92 ± 0,30	<0,001**
≤ 5	63	2,88 ± 0,80	
6–9	33	3,41 ± 0,82	
10–12	41	3,57 ± 0,85
> 12	22	3,69 ± 0,75
(n total)	169	
HbA1c (%)			
< 7	35	3,41 ± 0,80	0,134*
≥ 7	130	3,15 ± 0,94	
(n total)	165		
Glicemia de Jejum (mg/dL)			
≤ 130	60	3,33 ± 0,92	0,201*
> 130	105	3,14 ± 0,91	
(n total)	165		

*Teste t de Student para amostras independentes ou teste não-paramétrico de Mann-Whitney, p < 0,05;

**ANOVA com um fator ou teste não-paramétrico de Kruskal-Wallis, p < 0,05.

A Tabela 4 mostra a correlação fraca da escolaridade e da renda familiar com a subescala 9 do HLQ-Br (*r* = 0,50 e 0,35, respectivamente) e valor significativo com a HbA1c (*p* = 0,031).

**Tabela 4:** Correlação das subescalas 6 e 9 do *Health Literacy Questionnaire* com as variáveis sociodemográficas e clínicas dos adultos com Diabetes *Mellitus* Tipo 2 – Região Metropolitana de Curitiba, PR, Brasil, 2024.

Variáveis	N	r	p-valor*
Subescala 6			
Idade	169	-0,001	0,985
Escolaridade	169	0,04	0,600
Renda familiar	168	0,12	0,123
Tempo diagnóstico	169	0,01	0,907
HbA1c	165	-0,07	0,364
Glicemia jejum	165	-0,09	0,230
Subescala 9			
Idade	169	-0,10	0,184
Escolaridade	169	**0,50**	**< 0,001**
Renda familiar	168	**0,35**	**< 0,001**
Tempo diagnóstico	169	-0,09	0,243
HbA1c	165	-0,17	**0,031**
Glicemia jejum	165	-0,05	0,518

*Coeficientes de Correlação de Spearman, p < 0,05.

Ao analisar o coeficiente alfa de Cronbach das subescalas relacionadas à capacidade de interagir ativamente com os profissionais de saúde e compreender as informações sobre saúde e saber o que fazer, obtiveram-se os valores de 0,82 e 0,74, respectivamente, considerados satisfatórios.

## DISCUSSÃO

Os resultados deste estudo revelam a relação significativa entre as variáveis sociodemográficas e clínicas com as duas subescalas do HLQ-Br. Verificou-se que os homens e aqueles com menores taxas de glicemia de jejum obtiveram maiores habilidades de LS no quesito de interação com os profissionais de saúde, podendo inferir que as orientações relacionadas à saúde viabilizaram a compreensão da importância do plano terapêutico e da adoção de hábitos de vida saudáveis na perspectiva do melhor monitoramento e eficácia das taxas glicêmicas.

Resultado similar foi encontrado em pesquisa realizada na África, que apresentou relação significativa da subescala 6 do HLQ e sexo masculino (*p* = 0,006)^(15)^. Por outro lado, pesquisa britânica não encontrou associação entre tais variáveis^(8)^. Estudo conduzido no Rio de Janeiro, Brasil, com 107 participantes acompanhados em ambulatório de DM, que utilizou a *European Health Literacy Survey*, por meio do modelo de regressão ordinal simples mostrou que os participantes do sexo masculino tinham três vezes maiores chances de ter nível de LS excelente (*p* = 0,03)^(16)^. Nesse sentido, acredita-se que diante das experiências masculinas do adoecimento crônico, há mobilização na busca por conhecimentos e suporte social junto aos serviços e profissionais de saúde, para o desenvolvimento de habilidades promotoras de bem-estar físico e mental^(17)^.

Destaca-se que a Política Nacional de Atenção Integral à Saúde do Homem (PNAISH), do MS brasileiro, publicada em 2009, enfatiza em seus objetivos a promoção de mudanças dos paradigmas relativos à percepção da população masculina sobre o cuidado com a saúde, bem como a qualificação dos profissionais da rede básica para o atendimento às demandas dessa população^(18)^. Todavia, pesquisa indicou que as ações de educação permanente e apoio da gestão dos cuidados aos homens ocorriam de forma incipiente e fragmentada, em decorrência da falta de capacitação e embasamento teórico-prático dos profissionais^(19)^. Resultados divergentes foram encontrados em estudo que evidenciou que os homens desconheciam as políticas de saúde e sentiam-se insatisfeitos com o atendimento recebido, pela falta de acolhimento profissional, de recursos materiais e de infraestrutura, e afirmavam que as mulheres recebiam mais atenção do que eles na APS^(20)^.

A associação entre a capacidade de interação com os profissionais da saúde e os níveis glicêmicos mostrou que os participantes com glicemia de jejum ≤ 130 mg/dL obtinham maiores habilidades de LS neste quesito, podendo inferir que se sentiam mais seguros para expor as suas experiências e condições de saúde, sem o receio de imposições ou julgamentos dos profissionais. Destarte, uma pesquisa australiana, que objetivou explorar o que os participantes com DM gostariam que seus profissionais de saúde entendessem sobre viver com a doença, resultou em percepções que indicavam a falta de atendimento centrado na pessoa, e que os julgamentos, as suposições e as perspectivas negativas dos profissionais não os ajudavam a gerenciar a doença^(21)^.

Os profissionais de saúde são corresponsáveis pelo desenvolvimento de habilidades de autogestão, por meio de ações de educação em saúde e de apoio, as quais são direcionadas às informações básicas para gerenciar a doença e os cuidados, no fortalecimento às mudanças de hábitos de vida e ao suporte psicossocial, reconhecendo a carga emocional de viver e do administrar a DM. As ações educativas oportunizam o conhecimento, a confiança e o aperfeiçoamento de habilidades para gerenciar os cuidados, no intuito de instrumentalizar a pessoa para a tomada de decisões e aumentar a resolutividade dos problemas^(22)^.

Neste sentido, reforça-se a importância da avaliação do LS em pessoas com DM2, na intenção de reconhecer a busca e as fontes de informações, a interpretação e o julgamento com relação aos cuidados e necessidades demandadas pela doença. Portanto, devem-se admitir as diferenças culturais e os determinantes sociais de saúde, a fim de promover uma assistência equitativa e centrada na pessoa^(22)^, elevando as habilidade de LS da população com DM2.

No que tange a associação da escolaridade com as habilidades de compreensão das informações de saúde e saber o que fazer, os participantes com menor tempo de educação formal apresentaram as menores médias de LS, quando comparados aos que tinham 12 anos ou mais de ensino. Pode-se deduzir que a falta e/ou incompletude de escrita e leitura dos participantes comprometem a obtenção e interpretação das informações de saúde, dificultando o julgamento perante adversidades impostas pelo adoecimento. Esses fatores repercutem negativamente na vida da pessoa com DM2, pois a falta do entendimento global da doença pode levar ao risco de complicações, à piora da qualidade de vida e ao aumento do número de hospitalizações e despesas onerosas ao indivíduo e ao sistema de saúde^(23)^.

Reforça-se que mais da metade da população deste estudo (n = 106) possuía menos de 10 anos de escolaridade, resultado similar à pesquisa realizada em Burkina Faso, na África Ocidental, que obteve maior concentração de participantes com educação primária (n = 175; 77,7%), sendo essa variável associada a baixa habilidade de compreensão das informações de saúde e sobre saber o que fazer, pela aplicação do HLQ (*p* = 0,004)^(15)^.

Outra pesquisa, conduzida na Espanha com 252 participantes diagnosticados com doenças cardiovasculares, encontrou associação da subescala 9 do HLQ com o nível de escolaridade (*p =* <0,001) e classe social (*p =* <0,001), sendo que a maior concentração dos participantes era de nível educacional básico (50,0%) e de classe social média (81,7%)^(24)^. Embora neste estudo não se tenha obtido significância estatística na associação entre a subescala 9 do HLQ com a renda familiar, verificou-se uma fraca correlação da capacidade de compreensão das informações de saúde e saber o que fazer com as variáveis escolaridade, renda familiar e HbA1c.

Pontua-se que pessoas com baixo nível de escolaridade tendem a maiores dificuldades para inserção no mercado de trabalho formal, ocasionando a precarização da ocupação, principalmente no que diz respeito à remuneração, que consequentemente os coloca em condição de pobreza^(25)^. Nesta vertente, ao considerar os baixos níveis de escolaridade e renda, e altas taxas glicêmicas dos participantes deste estudo, reflete-se sobre o saber o que fazer, em decorrência das barreiras financeiras para acessar produtos dietéticos, que auxiliam no controle glicêmico, a exemplo dos alimentos fontes de fibras, como as frutas e verduras, os laticínios e carnes magras^(26)^. Infere-se que outro entrave, relacionado ao nível da escolaridade, diz respeito à compreensão de orientações e à interpretação adequada de rótulos de alimentos para escolhas mais saudáveis.

Em pesquisa transversal com 33 participantes com DM2, verificou-se fraca correlação entre os níveis de glicemia de jejum e a renda familiar (*r*= 0,1946) e correlação moderada com o conhecimento sobre a doença (*r* = 0,4260), indicando que quanto maior a renda familiar, menor eram os valores glicêmicos e melhor era a compreensão sobre a DM^(27)^. Diante dessas condições, os profissionais da APS devem adequar suas formas de comunicação e linguagem, e os serviços e sistemas de saúde devem ser aperfeiçoados, a fim de oferecer subsídios e ferramentas para minimizar a discrepância relacionada ao LS^(28)^, principalmente das pessoas com baixos níveis de escolaridade e renda.

Nesta vertente, as ações de educação em saúde, embasadas no LS, são estratégias que possibilitam o desenvolvimento de habilidades promotoras de conhecimentos e autoeficácia. Os efeitos dessas estratégias foram verificados em ensaio clínico randomizado com 107 participantes com DM, dos quais 54 pertenceram ao grupo intervenção, que recebeu aconselhamentos por telefone. Após o período de seis meses, os participantes do grupo intervenção com baixos níveis de LS obtiveram melhora significativa na autoeficácia (*p* = 0,000), assim como aqueles com altos níveis (*p* = 0,016)^(29)^. Desse modo, infere-se que a utilização de ações educativas voltadas ao LS pode auxiliar no nivelamento das habilidades interpretativas e decisórias, principalmente no entendimento da complexidade relacionada ao gerenciamento da DM.

A aplicabilidade das subescalas 6 e 9 do HLQ-Br para a população adulta com DM2 mostrou boa consistência e os valores pelo Coeficiente de alfa de Cronbach foram similares aos obtidos na validação do instrumento para o português do Brasil, sendo de 0,78 para a subescala capacidade de interagir com os profissionais de saúde e de 0,76 para a compreensão das informações sobre saúde e saber o que fazer^(13)^.

Este estudo apresentou algumas limitações que devem ser consideradas ao interpretar os resultados. Primeiramente, a presença de acompanhantes durante a aplicação do questionário, o que pode ter afetado a interpretação das questões, principalmente quando realizado na forma de entrevista. A amostra, embora representativa, pode não refletir adequadamente a diversidade da população de adultos com DM2, limitando a generalização dos achados para contextos mais amplos. Há que se considerar que os estudos sobre LS em DM apresentam limitações metodológicas significativas, em razão da tipologia transversal e da falta de padronização nas ferramentas de mensuração, o que dificulta a comparação entre pesquisas^(23)^.

Os fatores externos, como a situação socioeconômica, as políticas e os programas de saúde em vigor no país, podem ter influenciado os níveis de LS, a exemplo do modelo de financiamento de recursos do Sistema Único de Saúde (SUS), o Programa Previne Brasil, que dentre os seus critérios apresentava o pagamento por desempenho com o objetivo de ampliar os atendimentos e acompanhar periodicamente os usuários com DM. Todavia, no ano de 2024, o programa passou por modificações significativas com a implementação de novos componentes de financiamento^(30)^. Tais considerações são importantes na interpretação dos resultados e para futuras pesquisas na área de LS entre adultos com DM2.

## CONCLUSÃO

Conclui-se que houve relação entre capacidade de interação com os profissionais de saúde e sexo masculino, compreendendo que os homens possuíam maior criticidade para os cuidados à saúde, na busca por informações e questionamentos aos profissionais, assim como aqueles que apresentavam baixas taxas de glicemia de jejum. Encontrou-se correlação fraca entre compreender as informações de saúde e saber o que fazer, renda familiar, escolaridade e HbA1c, resultados que podem contribuir para o desenvolvimento de ações educativas voltadas ao fortalecimento do LS.

Acredita-se que a implementação de programas de educação permanente aos profissionais da APS, com foco na adequação de linguagem simples, possa otimizar a interação e a compreensão das informações aos usuários dos serviços de saúde com DM2, em especial as mulheres, aqueles com baixos níveis de escolaridade e com taxas de glicemia de jejum > 130 mg/dL, a fim de reduzir as complicações relacionadas à doença e capacitar as pessoas para a autogestão dos cuidados. Além disso, é fundamental que políticas públicas sejam desenvolvidas para abordar as desigualdades do LS, especialmente em populações com menor escolaridade e renda.

Incentiva-se que futuras pesquisas explorem intervenções específicas que visem melhorar as habilidades de LS e avaliar sua repercussão nas condições de saúde e na qualidade de vida da população. Essas iniciativas são essenciais para promover a assistência mais equitativa e centrada na pessoa, contribuindo para a mitigação das complicações associadas ao DM2, e para o fortalecimento da autonomia e da promoção da saúde.

# Translation: Alfabetización en salud de adultos con diabetes *mellitus* tipo 2 en la atención primaria: estudio seccional

**DOI:** 10.1590/1980-220X-REEUSP-2024-0338es

**Authors:** Robson Giovani Paes, Isabella Bueno Fusculim, Luciane Lachouski, Ingrid Marcela Pinto Gariba de Andrade, Shirley Boller, Maria de Fátima Mantovani

**Affiliations:** 1 Universidade Federal do Paraná, Curitiba, PR, Brasil.

**Keywords:** Enfermería, Diabetes Mellitus, Alfabetización en Salud, Atención Primaria de Salud

## Abstract

**Objetivo:**

Relacionar la alfabetización en salud de los adultos con diabetes mellitus tipo 2 provenientes de la atención primaria de salud con variables sociodemográficas y clínicas.

**Método:**

Estudio descriptivo analítico transversal con 169 participantes de 18 a 65 años con diabetes mellitus tipo 2, de seis unidades de atención primaria de la Región Metropolitana de Curitiba, Paraná, Brasil. La recolección de datos se realizó de julio de 2023 a marzo de 2024, y se utilizó un cuestionario sociodemográfico y clínico, así como las subescalas seis y nueve del *Cuestionario de alfabetización en salud* Versión brasileña. Los datos fueron analizados descriptivamente y mediante prueba t de Student, pruebas de Mann-Whitney, Anova, Kruskal-Wallis y Spearman.

**Resultados:**

De los 169 participantes, la edad media fue de 56,5 ± 6,2 años. Se encontró una asociación significativa entre la capacidad de interactuar con profesionales de la salud y el género masculino (*p* = 0,057), glucemia en ayunas ≤130 mg/dl (*p* = 0,033), y entre la educación y la comprensión de la información sanitaria y saber qué hacer (*p* = <0,001). Se encontró una correlación débil entre la comprensión de la información de salud y saber qué hacer y la educación y los ingresos familiares (*a* = 0,50 y 0,35, respectivamente) y valor significativo con hemoglobina glucosilada (*p* = 0,031).

**Conclusión:**

Se encontró una relación entre la alfabetización en salud y el género masculino, la educación, el ingreso familiar y los valores glucémicos. Estos resultados pueden contribuir al desarrollo de acciones educativas dirigidas a fortalecer la alfabetización en salud.

## INTRODUCCIÓN

La alfabetización en salud (AS) es un posible determinante social de la salud^(1)^ que expresa el grado en que las personas son capaces de recibir, procesar y comprender la información y los servicios básicos necesarios para tomar decisiones adecuadas sobre su estado de salud^(1,2)^. Se define como el conocimiento, la motivación y las habilidades para acceder, evaluar y aplicar la información en el ámbito de la prevención de enfermedades, la asistencia y la promoción de la salud con el fin de mejorar o mantener la calidad de vida y la autonomía en la toma de decisiones^(2)^.

La AS también puede conceptualizarse como un conjunto de habilidades individuales y sociales que, mediante el intercambio de información entre profesionales, usuarios y sistemas, refuerzan el juicio oportuno para la toma de decisiones en salud^(3)^. Analizando los niveles de AS, es posible identificar el grado de implicación del individuo en el proceso salud-enfermedad, su reconocimiento de los factores de riesgo, el acceso a la información y a los servicios, y la posibilidad de crear acciones de participación activa en el cuidado de la salud^(4)^.

El análisis de AS está en consonancia con las medidas de prevención y vigilancia de las enfermedades crónicas no transmisibles presentadas en el plan de acción estratégico del Ministerio de Salud de Brasil, que defiende la importancia de que las personas conozcan los factores de riesgo, tengan acceso a bienes y servicios públicos y se garanticen derechos de información que permitan opciones favorables en materia de salud^(5)^.

Al considerar las enfermedades crónicas no transmisibles, la diabetes *mellitus* (DM) se destaca como un importante problema de salud pública debido a su prevalencia y complicaciones^(6)^. La diabetes *mellitus* tipo 2 (DM2) representa alrededor del 90–95% de los casos de la enfermedad y sus factores desencadenantes son los antecedentes familiares, la dificultad para controlar el peso y los hábitos de vida sedentarios, siendo responsable de altas tasas de complicaciones micro y macrovasculares como accidentes cerebrovasculares, infarto agudo de miocardio, amputaciones, retinopatía, neuropatía y otros problemas^(6)^.

En 2021, Brasil *ocupaba el* sexto lugar en el ranking mundial de la enfermedad, con aproximadamente 15 millones de personas con DM en el grupo de edad de 20 a 79 años, con un aumento proyectado de 7,5 millones de nuevos casos para 2045^(7)^. Esta proyección se torna preocupante en presencia de complicaciones típicamente comunes a la DM2, que pueden estar relacionadas con bajos niveles de AS y falta de conocimiento sobre la enfermedad, lo que puede interferir directamente en la obtención y comprensión de información sobre salud^(4)^. Estos aspectos fueron señalados en un estudio anterior realizado con 33 adultos diagnosticados con DM2, registrados en una unidad de salud de la región sur de Brasil, a quienes se les aplicaron los instrumentos *Conocimiento Hablado en Pacientes con Diabetes con Bajo Nivel de Alfabetización* (*Spoken Knowledge in Low Literacy Patients with Diabetes* – SKILLD) y *Herramienta de Evaluación de Alfabetización en Salud de Ocho Ítems* (*Eight-Item Health Literacy Assessment Tool* – HLAT-8) para analizar el conocimiento sobre la enfermedad y la AS. Tras la intervención educativa de enfermería, se produjo un aumento de los conocimientos sobre DM (*p* = 0,001) correlacionado con la AS (*p* = 0,001)^(4)^, y puede inferirse que la interacción profesional y las acciones centradas en la persona ayudaron a los participantes a buscar y comprender la información y los servicios sanitarios.

Teniendo esto en cuenta, se intentó comprender la capacidad de los adultos con DM2 para interactuar con los profesionales de la salud, comprender la información sanitaria y saber qué hacer, a partir de un estudio británico con 2.309 participantes, en el que se relacionaron las subescalas seis y nueve del *Cuestionario de Alfabetización em Salud* (*Health Literacy Questionnaire* – HLQ), relativas a las habilidades de interacción con los profesionales y la comprensión de la información sanitaria, con variables sociodemográficas y clínicas. Los resultados mostraron que el 19,4% de los participantes tenía dificultades para leer y comprender la información sanitaria escrita y el 23,2% tenía dificultades para comentar sus preocupaciones con los profesionales sanitarios debido a su bajo estatus social y educativo. Cabe señalar que los participantes padecían al menos una enfermedad crónica^(8)^.

Por lo tanto, es fundamental identificar las barreras de comunicación, ya sean debidas al lenguaje técnicamente complejo utilizado por el profesional sanitario o a la información incorrecta o incompleta captada por el interlocutor, factores que pueden interferir en el cumplimiento de las intervenciones sanitarias, en el empeoramiento del cuadro clínico^(9)^ y en el desarrollo de las habilidades de AS. Se ha revelado que las personas con DM2 con altos niveles de AS tienden a adherirse mejor al tratamiento farmacológico y a los cambios en el estilo de vida, con un resultado en términos de control glicémico y mitigación de las complicaciones asociadas a la enfermedad^(10)^.

En este sentido, con el objetivo de contribuir a la atención integral de las personas con DM2 en el contexto de la Atención Primaria de Salud (APS), en función del grado de AS, este estudio tuvo como objetivo relacionar la alfabetización en salud de adultos con diabetes *mellitus* tipo 2 en atención primaria de salud con variables sociodemográficas y clínicas.

## MÉTODO

### Diseño del Estudio

Estudio cuantitativo, descriptivo, analítico, transversal, que siguió las recomendaciones del *Strengthening the Reporting of Observational studies in Epidemiology* (Strobe)^(11)^.

### Localización, Población y Criterios de Selección

El estudio fue realizado en seis unidades de APS localizadas en la Región Metropolitana de Curitiba, Paraná, Brasil. El municipio estaba entre las diez ciudades más pobladas del estado, con aproximadamente 232.212 habitantes y un Índice de Desarrollo Humano Municipal (IDHM) de 0,733, considerado similar al del país. Su distribución territorial era de 197,580 km^2^ con una densidad poblacional de 1.175,28 hab/km^2(12)^.

En el ámbito de la salud, el municipio contaba con 25 unidades de APS, organizadas en tres distritos de salud, con cerca de 11.278 usuarios diagnosticados con DM e inscritos en el programa de hipertensión y DM del Ministerio de Salud (Hiperdia).

Los criterios de elegibilidad fueron usuarios de los servicios de salud de entre 18 y 65 años, inscritos en el programa Hiperdia y con diagnóstico confirmado de DM2. Se excluyeron los usuarios con comorbilidades cognitivas y/o de comunicación, identificadas por la descripción en la historia clínica electrónica.

### Definición de la Muestra y Reclutamiento

El estudio forma parte de un proyecto más amplio que utilizó un muestreo probabilístico poblacional por conglomerados. La muestra se planificó en base al número estimado de habitantes del municipio, considerando una tasa de prevalencia nacional de DM del 10,5% (intervalo de confianza [IC] del 95%: 9,4% a 11,6%), un error aceptable del 5% y una muestra heterogénea (50/50). El número de participantes por conglomerado se definió en proporción al número total de usuarios registrados con DM en cada unidad, y en este subproyecto, los datos se refieren al 25% (seis unidades de APS), dos de cada distrito sanitario.

La selección de los participantes se realizó mediante una aplicación de sorteo en línea, utilizando los nombres de todos los usuarios que figuraban en las listas del Programa Hiperdia facilitadas por los coordinadores de las unidades de APS. Este procedimiento garantizó la aleatoriedad de la muestra, permitiendo el acceso posterior a las historias clínicas para identificar y definir a los usuarios elegibles. Las listas se organizaron con la inclusión de suplentes para sustituir a los voluntarios en caso de negativas o falta de contactos.

Los usuarios aleatorizados fueron reclutados al mismo tiempo que se recogían los datos, mediante llamadas telefónicas y/o mensajes de texto estándar enviados a través de la aplicación WhatsApp®️. Se realizaron hasta tres intentos de contacto en días y horas diferentes para invitar a los usuarios a participar en el estudio. Si aceptaban, se concertaba una cita con enfermería, que se realizaba en la unidad de salud o en el domicilio, según la disponibilidad y preferencia del participante. En total, se contactó con 596 usuarios: 381 no contestaron a las llamadas o no respondieron a los mensajes, 46 se negaron a participar y 169 constituyeron la muestra final.

### Instrumentos de Recogida de Datos

Se utilizaron dos instrumentos para la recogida de datos: un cuestionario que contenía variables sociodemográficas y clínicas y el HLQ versión brasileña (HLQ-Br). El cuestionario contenía variables sociodemográficas (edad, sexo, escolaridad, estado civil e ingresos familiares) y clínicas (tiempo transcurrido desde el diagnóstico de DM2, hemoglobina glicosilada (HbA1c) y pruebas de glicemia en ayunas).

El HLQ-Br es un instrumento multidimensional desarrollado en Australia, que puede autoadministrarse o aplicarse en forma de entrevista. Contiene 44 preguntas divididas en nueve subescalas: 1 – Comprensión y apoyo de los profesionales de la salud; 2 – Información suficiente para cuidar de la salud; 3 – Cuidado activo de la salud; 4 – Apoyo social para la salud; 5 – Evaluación de la información sobre salud; 6 – Capacidad para interactuar activamente con los profesionales de la salud; 7 – Navegación por el sistema de salud; 8 – Capacidad para encontrar buena información sobre salud; y 9 – Comprensión de la información sobre salud y saber qué hacer. Ha sido validado para el portugués brasileño^(13,14)^.

El HLQ-Br es una herramienta que ayuda a identificar las condiciones de AS. Para este estudio, se seleccionaron las subescalas 6 y 9, que abordan cuestiones relacionadas con la comunicación con los profesionales de la salud y la comprensión y seguimiento de las instrucciones sanitarias. Las respuestas a estas subescalas siguen un formato Likert de cinco puntos, que van desde «siempre difícil (1)» a «siempre fácil (5)». La puntuación del HLQ se calculó a partir de la media de los ítems de cada subescala.

### Recogida de Datos

La recogida de datos tuvo lugar durante una consulta de enfermería, de una duración media de 50 minutos, realizada por la investigadora principal y 10 asistentes de investigación, que habían recibido una formación previa de cinco horas a través de un curso teórico-práctico sobre AS y DM.

Se administró un cuestionario sociodemográfico y clínico con las variables edad, sexo, escolaridad, estado civil, renta familiar y tiempo transcurrido desde el diagnóstico de DM2, que se obtuvieron por autodeclaración. Cabe señalar que un participante no pudo o no quiso declarar sus ingresos familiares mensuales.

Los valores de las pruebas de HbA1c y glicemia en ayunas se obtuvieron de las historias clínicas de los participantes y eran válidos hasta un año antes del estudio. En los casos en que las variables bioquímicas estaban desactualizadas, las pruebas se solicitaron a laboratorios acreditados por el departamento de salud del municipio, a través del Programa Previne Brasil del Ministerio de Salud. Cabe señalar que se pidió a 25 participantes que se hicieran las pruebas, pero cuatro no lo hicieron, lo que redujo el tamaño de la muestra para esta variable.

Las dos subescalas del HLQ-Br fueron aplicadas en aproximadamente 20 minutos. Para los participantes con baja visión y dificultades de lectura, el instrumento se aplicó en forma de entrevista.

### Análisis y Procesamiento de los Datos

Los datos fueron introducidos en hojas de cálculo *de Microsoft Excel®*por dos investigadores de forma independiente. Las dos hojas de cálculo fueron cotejadas entre sí por un profesional de la estadística para comprobar si había incoherencias, y los datos se analizaron utilizando el programa informático IBM® *Statistical Package for Social Sciences* (SPSS) v.29.0.0. El análisis de las variables cuantitativas descriptivas (edad, escolaridad, ingresos familiares, tiempo transcurrido desde el diagnóstico, valores de HbA1c y glicemia en ayunas) se presentó mediante media y desviación estándar (DE).

Las variables categóricas se describieron por frecuencia absoluta y porcentaje, como sigue: edad (45-59; 60-65 años), sexo (masculino/femenino), estado civil (soltero/casado/separado o divorciado/viudo), escolaridad (no alfabetizado; ≤ 5; 6–9; 10–12 y > 12 años), ingresos familiares (< 1. 320; 1.320–3.960; > 3.960 reales al mes), tiempo transcurrido desde el diagnóstico (< 1; 1–5; 6–10 y > 10 años), HbA1c (< 7 y ≥ 7%) y glicemia en ayunas (≤ 130 y > 130 mg/dL).

Se utilizó la prueba t de Student para muestras independientes o la prueba no paramétrica de Mann-Whitney para analizar la asociación entre las subescalas del cuestionario HLQ-Br y variables categóricas sociodemográficas y clínicas con dos clasificaciones. Las variables con más de dos clasificaciones se analizaron mediante el modelo de análisis de la varianza (ANOVA) de un factor o la prueba no paramétrica de Kruskal-Wallis. Se estimaron los coeficientes de correlación de Spearman (*r*) para analizar la correlación entre las subescalas y las variables cuantitativas. Los valores de correlación se interpretaron como excelentes (*r =* > 0,90), buenos (*r =* 0,75 a 0,90), moderados (*r =* 0,50 a 0,74) y débiles (*r =* < 0,50). Los valores de p < 0,05 indicaron significación estadística.

Los resultados de las subescalas 6 y 9 del HLQ-Br se presentaron como medidas puntuales y estimadas, con un IC del 95%. La fiabilidad de las subescalas para la población adulta con DM2 se comprobó mediante el coeficiente alfa de Cronbach (α), con un valor mínimo de 0,70.

### Aspectos Éticos

El estudio fue aprobado por el Comité de Ética de Investigación en Seres Humanos de la Universidad Pública del Estado de Paraná bajo el dictamen fundamentado no. 6.138.731 y CAAE 68471723.6.0000.0102. La investigación se llevó a cabo de acuerdo con los requisitos del Consejo Nacional de Salud (CNS) establecidos en la Resolución 466/2012.

El HLQ fue desarrollado por la Swinburne University of Technology, en Australia, que posee los derechos de propiedad intelectual del material. Para su aplicabilidad en este estudio, se obtuvo el código de licencia L23009IS y se siguieron las recomendaciones de los autores para la presentación de las preguntas del instrumento^(14)^.

## RESULTADOS

De los 169 (100%) participantes con DM2, 58,0% (n = 98) eran mujeres, con edad entre 40 y 65 años (56,5 ± 6,2), renta familiar promedio de R$ 4.032 ± 3.021 y 37,3% (n = 63) con cinco años o menos de escolaridad y 75,1% (n = 127) eran casadas y/o en unión consensual. En cuanto a las variables clínicas, 78,9% (n = 130) tenían valores de HbA1c ≥ 7%, 63,6% (n = 105) glicemia en ayunas > 130 mg/dL y 46,7% (n = 79) habían sido diagnosticados de DM2 hacía más de 10 años (Tabla 1).

**Tabla 1:** Caracterización sociodemográfica y clínica de adultos con Diabetes *Mellitus* Tipo 2 – Región Metropolitana de Curitiba, PR, Brasil, 2024.

Variables	n (%)	Media ± DE
Edad (años)		
40–59	106 (62,7)	56,5 ± 6,2
60–65	63 (37,3)	
(n total)	169 (100)	
Sexo		
Mujer	98 (58,0)	
Hombre	71 (42,0)	
(n total)	169 (100)	
Estado civil		
Único	15 (8,9)	
Unión matrimonial/consensual	127 (75,1)	
Separado/divorciado	19 (11,2)	
Viuda	8 (4,7)	
(n total)	169 (100)	
Escolaridad (años)		
No sabe leer ni escribir	10 (5,9)	7,6 ± 4,7
≤ 5	63 (37,3)	
6–9	33 (19,5)	
10–12	41 (24,3)	
> 12	22 (13,0)	
(n total)	169 (100)	
Ingresos familiares (reales)*		
< 1,320	17 (10,1)	4,032 ± 3,021
1.320–3.960	78 (46,4)	
> 3,960	73 (43,5)	
(n total)	168 (100)	
Tiempo transcurrido desde el diagnóstico (años)		
< 1	1 (0,6)	11,89 ± 8,6
1–5	48 (28,4)	
6–10	41 (24,3)	
> 10	79 (46,7)	
(n total)	169 (100)	
HbA1c (%)		
< 7	35 (21,2)	8,7 ± 2,1
≥ 7	130 (78,8)	
(n total)	165 (100)	
Glicemia en ayunas (mg/dL)		
≤ 130	60 (36,4)	173,6 ± 81,7
> 130	105 (63,3)	
(n total)	165 (100)	

DE = Desvío estándard.

*Se refiere al salario mínimo brasileño en julio de 2023.

La AS media en la subescala 6, es decir, la capacidad de interactuar activamente con los profesionales de la salud, fue de 3,67 ± 0,88 (IC 95%: 3,53–3,80) y mostró una asociación significativa con los varones (p= 0,057) y aquellos con valores de glicemia en ayunas ≤ 130mg/dL (p = 0,033) (Tabla 2).

**Tabla 2:** Asociación entre la capacidad de interactuar activamente con profesionales de salud (Subescala 6 – del *Cuestionario de Alfabetización en Salud – Health Literacy Questionnaire*) y las vari­ables sociodemográficas y clínicas de adultos con Diabetes *Mellitus* Tipo 2 – Región Metropolitana de Curitiba, PR, Brasil, 2024.

Variables	n	Media ± DE	p-valor
Edad (años)			
40–59	106	3,65 ± 0,88	0,684*
60–65	63	3,70 ± 0,89	
(n total)	169		
Sexo			
Mujer	98	3,56 ± 0,93	0,057*
Hombre	71	3,82 ± 0,80	
(n total)	169		
Escolaridad (años)			
No alfabetizado	10	3,24 ± 0,68	0,443**
≤ 5	63	3,71 ± 0,89	
6–9	33	3,70 ± 1,01	
10–12	41	3,77 ± 0,78
> 12	22	3,51 ± 0,91
(n total)	169	
HbA1c (%)			
< 7	35	3,85 ± 0,87	0,231*
≥ 7	130	3,64 ± 0,88	
(n total)	165		
Glicemia en ayunas (mg/dL)			
≤ 130	60	3,88 ± 0,81	0,033*
> 130	105	3,58 ± 0,90	
(n total)	165		

*Prueba t de Student para muestras independientes o prueba no paramétrica de Mann-Whitney, p < 0,05;

**ANOVA de una vía o prueba no paramétrica de Kruskal-Wallis, p < 0,05.

La media de la subescala 9, es decir, comprender la información sanitaria y saber qué hacer, fue de 3,20 ± 0,91 (IC 95%: 3,10–3,40), lo que muestra la asociación entre el nivel de educación y las habilidades LS (*p* = <0,001), siendo los participantes analfabetos y aquellos con ≤ 5 años de estudios formales los que presentaron medias y DE más bajas en la subescala (Tabla 3).

**Tabla 3:** Asociación entre comprender la información de salud y saber qué hacer (Subescala 9 – del *Cuestionario de Alfabetización en Salud – Health Literacy Questionnaire*) con las variables sociodemográficas y clínicas de adultos con Diabetes *Mellitus* Tipo 2 – Región Metropolitana de Curitiba, PR, Brasil, 2024.

Variables	n	Media ± DE	p-valor
Edad (años)			
40–59	106	3,24 ± 0,93	0,445*
60–65	63	3,13 ± 0,88	
(n total)	169		
Sexo			
Mujer	98	3,19 ± 0,99	0,903*
Hombre	71	3,21 ± 0,78	
(n total)	169		
Escolaridad (años)			
No sabe leer ni escribir	10	1,92 ± 0,30	<0,001**
≤ 5	63	2,88 ± 0,80	
6–9	33	3,41 ± 0,82	
10–12	41	3,57 ± 0,85
> 12	22	3,69 ± 0,75
(n total)	169	
HbA1c (%)			
< 7	35	3,41 ± 0,80	0,134*
≥ 7	130	3,15 ± 0,94	
(n total)	165		
Glicemia en ayunas (mg/dL)			
≤ 130	60	3,33 ± 0,92	0,201*
> 130	105	3,14 ± 0,91	
(n total)	165		

*Prueba t de Student para muestras independientes o prueba no paramétrica de Mann-Whitney, p < 0,05;

**ANOVA de una vía o prueba no paramétrica de Kruskal-Wallis, p < 0,05.

La Tabla 4 muestra la débil correlación de la escolaridad y la renta familiar con la subescala 9 del HLQ-Br (*r* = 0,50 y 0,35, respectivamente) y un valor significativo con la HbA1c (*p* = 0,031).

**Tabla 4:** Correlación de las subescalas 6 y 9 del *Cuestionario de Alfabetización en Salud (Health Literacy Questionnaire*) con las variables sociodemográficas y clínicas de adultos con Diabetes *Mellitus* Tipo 2 – Región Metropolitana de Curitiba, PR, Brasil, 2024.

Variables	N	r	Valor p*
Subescala 6			
Edad	169	-0,001	0,985
Educación	169	0,04	0,600
Ingresos familiares	168	0,12	0,123
Tiempo de diagnóstico	169	0,01	0,907
HbA1c	165	-0,07	0,364
Glicemia en ayunas	165	-0,09	0,230
Subescala 9			
Edad	169	-0,10	0,184
Educación	169	**0,50**	**< 0,001**
Ingresos familiares	168	**0,35**	**< 0,001**
Tiempo de diagnóstico	169	-0,09	0,243
HbA1c	165	-0,17	**0,031**
Glicemia en ayunas	165	-0,05	0,518

*Coeficientes de correlación de Spearman, p < 0,05.

Al analizar el coeficiente alfa de Cronbach de las subescalas relacionadas con la capacidad para interactuar activamente con los profesionales sanitarios y para comprender la información sanitaria y saber qué hacer, se obtuvieron los valores de 0,82 y 0,74, respectivamente, que se consideran satisfactorios.

## DISCUSIÓN

Los resultados de este estudio revelan una relación significativa entre las variables sociodemográficas y clínicas y las dos subescalas del HLQ-Br. Se observó que los hombres y las personas con niveles más bajos de glicemia en ayunas presentaban mayores habilidades de SA en cuanto a la interacción con los profesionales de la salud, pudiéndose inferir que la orientación sanitaria les permitía comprender la importancia del plan terapéutico y la adopción de hábitos de vida saludables con vistas a un mejor control y eficacia de los niveles de glucémicos.

Un resultado similar se encontró en un estudio realizado en África, que mostró una relación significativa entre la subescala 6 del HLQ y el sexo masculino (*p* = 0,006)^(15)^. Por otro lado, un estudio británico no encontró asociación entre estas variables^(8)^. Un estudio realizado en Río de Janeiro, Brasil, con 107 participantes seguidos en un ambulatorio de DM, en el que se utilizó el *European Health Literacy Survey*, mediante un modelo de regresión ordinal simple, mostró que los participantes varones tenían tres veces más probabilidades de tener un nivel excelente de AS (*p* = 0,03)^(16)^. En este sentido, se cree que frente a las experiencias masculinas de enfermedad crónica, hay movilización en la búsqueda de conocimiento y apoyo social de los servicios y profesionales de salud, para desarrollar habilidades que promuevan el bienestar físico y mental^(17)^.

Cabe destacar que la Política Nacional de Atención Integral a la Salud del Hombre (PNAISH) del Ministerio de Salud de Brasil, publicada en 2009, destaca en sus objetivos la promoción de cambios en los paradigmas relacionados con la percepción de la atención a la salud por parte de la población masculina, así como la cualificación de los profesionales de atención primaria para atender las demandas de esta población^(18)^. Sin embargo, un estudio indicó que la formación continuada y el apoyo a la gestión de la atención a los hombres eran incipientes y fragmentados, debido a la falta de formación y de bagaje teórico y práctico de los profesionales^(19)^. Se encontraron resultados divergentes en un estudio que mostró que los hombres desconocían las políticas de salud y se sentían insatisfechos con la atención que recibían, debido a la falta de acogida profesional, recursos materiales e infraestructura, y afirmaban que las mujeres recibían más atención que ellos en la APS^(20)^.

La asociación entre la capacidad de interactuar con los profesionales de salud y los niveles glicémicos mostró que los participantes con glicemia en ayunas ≤ 130 mg/dL tenían mayores habilidades de SA en este sentido, lo que puede inferir que se sentían más seguros para exponer sus experiencias y condiciones de salud, sin el temor de imposiciones o juicios de los profesionales. Así, un estudio australiano, cuyo objetivo era explorar qué les gustaría a los participantes con DM que entendieran sus profesionales sanitarios sobre cómo vivir con la enfermedad, dio como resultado percepciones que indicaban una falta de atención centrada en la persona, y que los juicios, suposiciones y perspectivas negativas de los profesionales no les ayudaban a manejar la enfermedad^(21)^.

Los profesionales de la salud son corresponsables del desarrollo de habilidades de autogestión a través de acciones de educación sanitaria y apoyo, dirigidas a proporcionar información básica para el manejo de la enfermedad y los cuidados, reforzando los cambios en los hábitos de vida y el apoyo psicosocial, reconociendo la carga emocional que supone vivir y manejar la DM. Las actividades educativas proporcionan conocimientos, confianza y habilidades para gestionar los cuidados, con el fin de capacitar a las personas para tomar decisiones y aumentar la resolución de problemas^(22)^.

En este sentido, se refuerza la importancia de evaluar la AS en personas con DM2, con la intención de reconocer la búsqueda y las fuentes de información, interpretación y juicio en relación a los cuidados y necesidades que demanda la enfermedad. Por lo tanto, las diferencias culturales y los determinantes sociales de la salud deben ser tenidos en cuenta para promover una atención equitativa y centrada en la persona^(22)^, aumentando las competencias en AS de la población con DM2.

Con respecto a la asociación entre la escolaridad y la capacidad de comprender la información de salud y saber qué hacer, los participantes con menor escolaridad formal tuvieron las puntuaciones promedio más bajas de AS en comparación con aquellos con 12 años o más de escolaridad. Se puede deducir que la falta y/o el carácter incompleto de las competencias de escritura y lectura de los participantes comprometen su capacidad para obtener e interpretar la información sanitaria, lo que les dificulta emitir juicios ante las adversidades impuestas por la enfermedad. Estos factores repercuten negativamente en la vida de las personas con DM2, ya que la falta de comprensión global de la enfermedad puede conllevar un riesgo de complicaciones, un empeoramiento de la calidad de vida y un aumento del número de hospitalizaciones y gastos costosos para el individuo y el sistema sanitario^(23)^.

Cabe destacar que más de la mitad de la población de este estudio (n = 106) tenía menos de 10 años de escolarización, un resultado similar al del estudio realizado en Burkina Faso, África Occidental, en el que se encontró una mayor concentración de participantes con estudios primarios (n = 175; 77,7%), asociándose esta variable con una baja capacidad para comprender la información sanitaria y saber qué hacer, mediante la aplicación del HLQ (*p* = 0,004)^(15)^.

Otro estudio, realizado en España con 252 participantes diagnosticados de enfermedad cardiovascular, halló una asociación entre la subescala 9 del HLQ y el nivel de estudios (*p =* <0,001) y la clase social (*p =* <0,001), con la mayor concentración de participantes con un nivel de estudios básico (50,0%) y clase social media (81,7%)^(24)^. Aunque en este estudio no se encontró una asociación estadísticamente significativa entre la subescala 9 del HLQ y la renta familiar, sí se observó una correlación débil entre la capacidad para comprender la información sanitaria y saber qué hacer y las variables de escolaridad, renta familiar y HbA1c.

Se ha señalado que las personas con bajo nivel de escolaridad tienden a tener mayores dificultades para insertarse en el mercado de trabajo formal, provocando la precarización de su ocupación, especialmente en términos de remuneración, lo que consecuentemente las coloca en condición de pobreza^(25)^. En este sentido, considerando los bajos niveles de escolaridad e ingresos, y los altos índices glicémicos de los participantes de este estudio, se reflexiona sobre el saber hacer, debido a las barreras financieras para acceder a productos dietéticos que ayudan al control glicémico, como alimentos fuentes de fibra, como frutas y verduras, lácteos y carnes magras^(26)^. Se infiere que otro obstáculo, relacionado con el nivel de escolarización, se refiere a la comprensión de las directrices y a la interpretación adecuada de las etiquetas de los alimentos para realizar elecciones más saludables.

En un estudio transversal de 33 participantes con DM2, hubo una correlación débil entre los niveles de glicemia en ayunas y el ingreso familiar (*r* = 0,1946) y una correlación moderada con el conocimiento sobre la enfermedad (*r* = 0,4260), lo que indica que cuanto mayor es el ingreso familiar, menores son los valores de glicemia y mejor es la comprensión de la DM^(27)^. Dadas estas condiciones, los profesionales de APS deben adaptar sus formas de comunicación y lenguaje, y los servicios y sistemas de salud deben ser mejorados para ofrecer subsidios y herramientas que minimicen la discrepancia relacionada con la AS^(28)^, especialmente entre las personas con bajos niveles de educación e ingresos.

En este sentido, las acciones de educación en salud basadas en AS son estrategias que permiten el desarrollo de habilidades que promueven el conocimiento y la autoeficacia. Los efectos de estas estrategias se comprobaron en un ensayo clínico aleatorizado con 107 participantes con DM, 54 de los cuales pertenecían al grupo de intervención, que recibió asesoramiento telefónico. Al cabo de seis meses, los participantes del grupo de intervención con niveles bajos de AS presentaron una mejora significativa de la autoeficacia (*p* = 0,000), al igual que los que tenían niveles altos (*p* = 0,016)^(29)^. De esta forma, se puede inferir que el uso de acciones educativas dirigidas a la AS puede ayudar a nivelar las habilidades interpretativas y de toma de decisiones, especialmente en la comprensión de la complejidad relacionada con el manejo de la DM.

La aplicabilidad de las subescalas 6 y 9 del HLQ-Br a la población adulta con DM2 mostró buena consistencia y los valores del coeficiente alfa de Cronbach fueron similares a los obtenidos en la validación del instrumento para el portugués de Brasil, con 0,78 para la subescala de habilidad para interactuar con profesionales de salud y 0,76 para comprender información de salud y saber qué hacer^(13)^.

Este estudio tuvo algunas limitaciones que deben tenerse en cuenta al interpretar los resultados. En primer lugar, la presencia de acompañantes durante el cuestionario, que puede haber afectado a la interpretación de las preguntas, especialmente cuando se realiza en forma de entrevista. La muestra, aunque representativa, puede no reflejar adecuadamente la diversidad de la población de adultos con DM2, limitando la generalizabilidad de los hallazgos a contextos más amplios. Hay que tener en cuenta que los estudios sobre AS en DM tienen importantes limitaciones metodológicas debido a su tipología transversal y a la falta de estandarización en las herramientas de medición, lo que dificulta la comparación de los estudios^(23)^.

Factores externos, como la situación socioeconómica, las políticas de salud y los programas vigentes en el país, pueden haber influido en los niveles de AS, como el modelo de financiación del Sistema Único de Salud (SUS), el Programa Previne Brasil, que incluía entre sus criterios el pago por desempeño, con el objetivo de ampliar la atención y monitorear periódicamente a los usuarios con DM. Sin embargo, en 2024, el programa sufrió cambios significativos con la implementación de nuevos componentes de financiación^(30)^. Estas consideraciones son importantes para la interpretación de los resultados y para futuras investigaciones sobre AS entre adultos con DM2.

## CONCLUSIÓN

Se puede concluir que existía una relación entre la capacidad de interactuar con los profesionales sanitarios y el sexo masculino, entendiendo que los hombres eran más críticos con la atención sanitaria, buscaban información y hacían preguntas a los profesionales, así como aquellos con niveles bajos de glicemia en ayunas. Se encontró una correlación débil entre la comprensión de la información sanitaria y el saber qué hacer, la renta familiar, la escolaridad y la HbA1c, resultados que podrían contribuir al desarrollo de acciones educativas dirigidas a fortalecer de la AS.

Se considera que la implementación de programas de educación continua para los profesionales de la APS, con foco en la adaptación de un lenguaje simple, podría optimizar la interacción y la comprensión de la información por parte de los usuarios de los servicios de salud con DM2, especialmente las mujeres, aquellos con bajo nivel de escolaridad y niveles de glicemia en ayunas > 130 mg/dL, a fin de reducir las complicaciones relacionadas con la enfermedad y capacitar a las personas para autogestionar su cuidado. Además, es esencial que se desarrollen políticas públicas para abordar las desigualdades de la AS, especialmente en las poblaciones con menor nivel educativo e ingresos.

Se anima a que en futuras investigaciones se exploren intervenciones específicas dirigidas a mejorar las competencias en AS y se evalúen sus repercusiones en las condiciones de salud y la calidad de vida de la población. Estas iniciativas son esenciales para promover una atención más equitativa y centrada en la persona, contribuir a mitigar las complicaciones asociadas a la DM2 y fortalecer la autonomía y la promoción de la salud.
